# Essential role for a novel population of binucleated mammary epithelial cells in lactation

**DOI:** 10.1038/ncomms11400

**Published:** 2016-04-22

**Authors:** Anne C. Rios, Nai Yang Fu, Paul R. Jamieson, Bhupinder Pal, Lachlan Whitehead, Kevin R. Nicholas, Geoffrey J. Lindeman, Jane E. Visvader

**Affiliations:** 1 Stem Cells and Cancer Division, The Walter and Eliza Hall Institute of Medical Research, Parkville, Victoria 3052, Australia; 2 Department of Medical Biology, The University of Melbourne, Parkville, Victoria 3010, Australia; 3 Imaging Laboratory, Systems Biology and Personalised Medicine Division, The Walter and Eliza Hall Institute of Medical Research, Parkville, Victoria 3052, Australia; 4 Department of Anatomy and Developmental Biology, Monash University, Clayton, Victoria 3800, Australia; 5 Familial Cancer Centre and Department of Medical Oncology, The Royal Melbourne Hospital, Parkville, Victoria 3050, Australia; 6 Department of Medicine, The University of Melbourne, Parkville, Victoria 3010, Australia

## Abstract

The mammary gland represents a unique tissue to study organogenesis as it predominantly develops in the post-natal animal and undergoes dramatic morphogenetic changes during puberty and the reproductive cycle. The physiological function of the mammary gland is to produce milk to sustain the newborn. Here we view the lactating gland through three-dimensional confocal imaging of intact tissue. We observed that the majority of secretory alveolar cells are binucleated. These cells first arise in very late pregnancy due to failure of cytokinesis and are larger than mononucleated cells. Augmented expression of Aurora kinase-A and Polo-like kinase-1 at the lactogenic switch likely mediates the formation of binucleated cells. Our findings demonstrate an important physiological role for polyploid mammary epithelial cells in lactation, and based on their presence in five different species, suggest that binucleated cells evolved to maximize milk production and promote the survival of offspring across all mammalian species.

## Abstract (web summary)

The main function of the mammary gland is to produce milk to sustain offspring. Here, the authors show that secretory alveolar cells in the lactating gland in several species are binucleated, which increases milk production, and that binucleation is regulated by Aurora kinase-A and Polo-like kinase-1.

## Graphical abstract

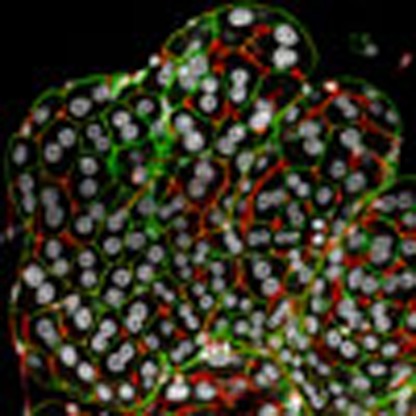

The overall structure of an organ is dictated by the shape, size and arrangement of its constituent cells. The mammary gland is a remarkably adaptive organ that offers a unique model to understand how an organ changes its structure to meet its physiological requirements. Post-natal development of the mammary gland occurs through distinct stages, encompassing puberty, pregnancy, lactation and involution, each of which involves drastic changes in tissue architecture^1^,^2^. Ductal morphogenesis in puberty culminates in the generation of a highly elaborate bilayered ductal tree comprising cells of the luminal and myoepithelial lineages. During pregnancy, the number of epithelial cells increases exponentially, with the formation of alveolar luminal units that differentiate in late pregnancy. Labelling assays have indicated that cell proliferation rapidly declines after mid-pregnancy as the gland commits to differentiation, and that a synchronized round of DNA synthesis occurs in early lactation^3^,^4^,^5^.

When alveolar epithelial cells enter the secretory activation phase in late pregnancy, they accumulate endoplasmic reticulum and golgi, and become enlarged through the production of milk. Milk contains several nutritional components for the newborn, including milk proteins, lipids and carbohydrates^6^,^7^. At the switch to lactation, the outer myoepithelial cells contract in response to oxytocin to expel milk from the alveolar luminal cells. Understanding the tissue remodelling processes that underlie the formation of the specialized milk-producing alveoli requires a comprehensive view of the cellular structure of the mammary gland.

In this report, we have applied three-dimensional (3D) imaging technology to provide a unique view of the mouse mammary gland and uncovered the presence of a large population of binucleated alveolar cells in lactation. These cells first appear in late pregnancy owing to failed cytokinesis rather than cell fusion. Mechanistically, we show that Aurora kinase-A (AURKA) and Polo-like kinase-1 (PLK-1) likely control the generation of binucleated cells at the switch to lactation, in response to signals that include prolactin and epidermal growth factor (EGF). The binucleated alveolar state was shown to be essential for effective lactation. Pertinently, polyploid cells were readily identified in the lactating mammary glands of four other mammalian species (human, cow, seal and wallaby), implying that polyploidy is an evolutionarily conserved mechanism to enable successful lactation. Our findings that binucleated cells are required for lactation represent one of the few physiological functions ascribed to polyploid mammalian cells thus far.

## Results

### A novel population of binucleated alveolar cells in lactation

**Figure 1:**
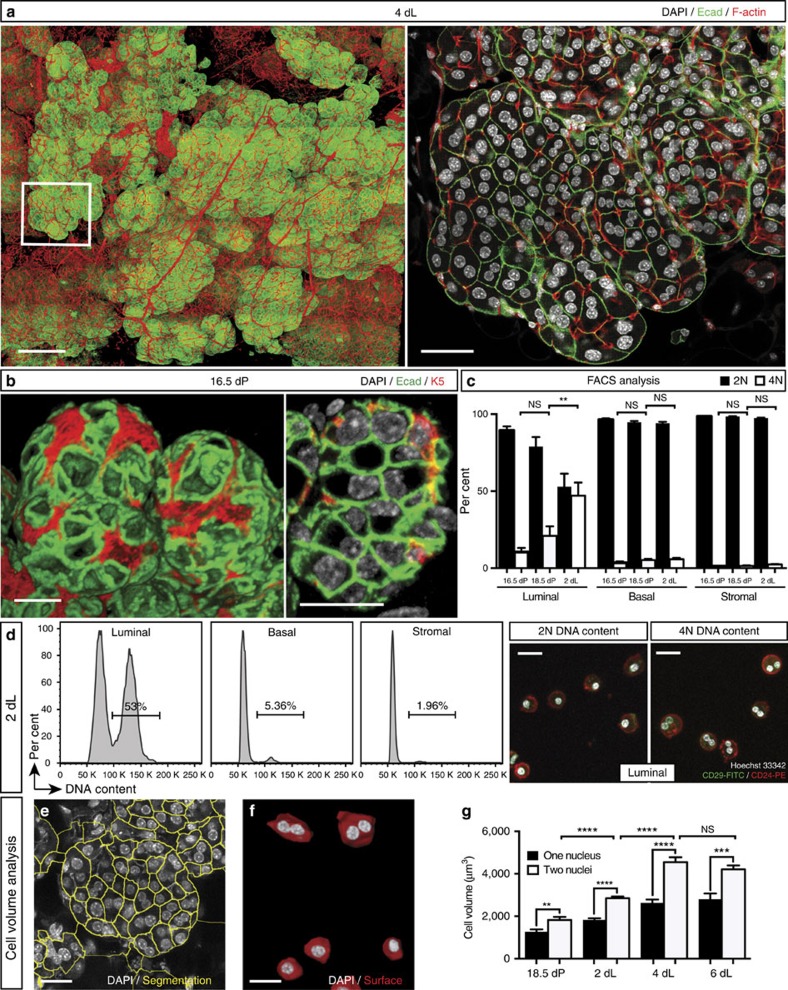
Identification and characterization of binucleated luminal cells in lactating glands. (**a**) Whole-mount 3D confocal image of a mammary ductal portion (FVB/N mouse) at 4 dL (left) and optical section of the enlarged region showing an alveolar unit (right). The gland was stained for DAPI (white), E-cadherin (Ecad; green), F-actin (red) and milk (blue, see Supplementary Fig. 1a) (*n*=3 mice). DAPI staining shows that the vast majority of luminal cells are binucleated. Scale bars: 200 μm (whole-mount); and 40 μm (optical section). (**b**) Whole-mount 3D confocal image of a mammary ductal portion (FVB/N mouse) at 16.5 dP (left) and optical section showing a representative alveolar unit (right). The gland was stained for DAPI (white), E-cadherin (green) and keratin 5 (red) (*n*=3 mice). No binucleated cells could be detected. Scale bars: 20 μm. (**c**) Bar graph showing the percentage of cells containing 2N or 4N DNA content in the luminal (Lin^−^CD29^lo^CD24^+^), basal (Lin^−^CD29^hi^CD24^+^) and stromal cell (Lin^−^CD24^−^) compartments in late pregnancy (16.5 and 18.5 dP) or early lactation (2 dL). Error bars represent mean±s.e.m. (*n*=3). (**d**) Representative FACS plots of DNA ploidy in the luminal, basal and stromal populations at 2 dL, and confocal images of sorted cells from the luminal population with 2N or 4N DNA content (far right panels). (**e**) Optical section from a 3D image of a mammary gland at 4 dL to illustrate the first step in volume quantification. Using ImageJ software, the cell membranes were outlined (yellow) based on E-cadherin immunostaining. Scale bar: 20 μm. (**f**) 3D image of representative bi- and mononucleated cells used for volume quantification performed with Imaris software. Scale bar: 20 μm. (**g**) Bar graph showing volume for luminal cells with either one or two nuclei at 18.5 dP, 2 dL, 4 dL and 6 dL (*n*=3 samples per time-point). Error bars represent mean±s.e.m. **P<0.01, ***P<0.001, ****P<0.0001.

We previously developed a high resolution 3D confocal imaging technique to visualize expansive regions of intact tissue (up to 1 cm) at single-cell resolution^8^. Using this technology to study mammary gland architecture, we surprisingly unveiled a substantial fraction of binucleated luminal cells (E-cadherin^+^) in the lactating mammary gland (Fig. 1a, Supplementary Fig. 1a and Supplementary Movie 1). Although an early dissertation reported the presence of binucleated cells in lactation^9^, this observation was considered to possibly represent an artefact of tissue fixation^10^ and no further evidence for this phenomenon has been described over decades. This largely reflects the limitations of two-dimensional microscopy, where it is difficult to visualize cells in their native state, particularly in densely packed tissue such as the lactating mammary gland, thus necessitating the use of 3D confocal imaging. While a majority of binucleated luminal cells was readily identifiable in the lactating gland by this technology, these cells were not detectable on day 16.5 of pregnancy (Fig. 1b) but appeared by day 18.5 when the gland has entered the secretory phase (Supplementary Fig. 1b)^6^. No cells containing more than two nuclei were observed, indicating that this process is strictly regulated. Myoepithelial cells, resident in the sheath around the alveolar luminal cells, remained mononucleated at all stages of development. Immunostaining at 4 days of lactation indicated that binucleated cells expressed abundant milk protein (Supplementary Fig. 1a). At 18.5 days of pregnancy, the nuclei and organelles were localized to the cell periphery on either side of the large cytoplasmic lipid droplet that characterizes cells at this stage (Supplementary Fig. 2a,b). Curiously, the size and location of this droplet changes after parturition such that large lipid droplets are replaced by small droplets localized to the apical surface of alveolar cells^6^. Thus, in lactating tissue, the nuclei and organelles are positioned within the central area of the alveolar cells (Supplementary Fig. 2c).

Following the cessation of lactation, the number and differentiation status of epithelial cells returns to their pre-pregnant state^11^. Involution is an essential process to remove milk-producing cells that are no longer required and is executed through large-scale programmed cell death. Notably, binucleated cells appear highly susceptible to cell death and are rapidly cleared within the first 48 hours of involution by apoptosis (Supplementary Fig. 3c,b), as indicated by the presence of binucleated cells positive for cleaved caspase-3 (CC3). No CC3^+^ cells were evident in lactation, as expected (Supplementary Fig. 3a,b). Efficient apoptosis of polyploid cells during involution is envisaged to be crucial for returning the gland to its pre-pregnant state in order to avert cell transformation of polyploid cells that might occur in response to hormonally-driven oestrus cycling. Indeed, genome duplication has been shown to promote neoplastic transformation of mammalian cells^12^,^13^.

### Increased DNA ploidy of alveolar cells from late pregnancy

To establish the DNA ploidy of cells during pregnancy and lactation, we performed flow cytometric analysis of three different cell populations: the luminal (Lin^–^CD29^lo^CD24^+^), stem cell-enriched basal (Lin^–^CD29^hi^CD24^+^) and stromal (Lin^–^CD24^–^) subsets^14^. At 16.5 days of pregnancy, only a small proportion of alveolar luminal cells contained a 4N DNA content by flow cytometry (Fig. 1c). The proportion of these cells increased dramatically from ∼17% at 18.5 days of pregnancy to ∼50% at 2 days of lactation (Fig. 1c,d). The higher proportion of binucleated cells apparent by confocal analysis (Fig. 1a) presumably reflects the fragility of these cells, which can be lost during tissue dissociation. In contrast to the luminal compartment, the basal and stromal populations contained a small subset of cells (<8%) with a DNA content of 4N in pregnancy and lactation. To address the cycling status of mammary epithelial cells at 16.5 versus 18.5 days of pregnancy, Hoechst-stained cell populations were analysed by flow cytometry. A comparable percentage of cells exhibited a 4N DNA content (12.5 versus 17.4% at 16.5 and 18.5 days of pregnancy, respectively) (Supplementary Fig. 4). However, binucleated cells were only apparent at 18.5 days of pregnancy based on confocal analysis of sorted cells with either a 2N or 4N DNA content (Supplementary Fig. 4). Thus, the 4N peak at 18.5 days pregnancy (dP) represents cells in the binucleated state plus cells in the G2/M phase of the cell cycle, whereas the 4N peak at 16.5 days corresponds to cells in G2/M.

### Larger cell volume conferred by the binucleated state

We next performed a volumetric analysis of alveolar luminal cells from late pregnant and lactating mammary glands. In order to capture quantitative information from our complex 3D imaging data (up to 150 GB per image), we established a semi-automated technique (see Methods). Substantially greater volumes were observed for binucleated compared with mononucleated cells (Fig. 1e–g), but the sizes of individual nuclei were comparable. Notably, the overall cell volume increased substantially from late pregnancy to 4 days of lactation but plateaued after this time (Fig. 1g).

### Polyploid cells arise through failed cytokinesis

**Figure 2:**
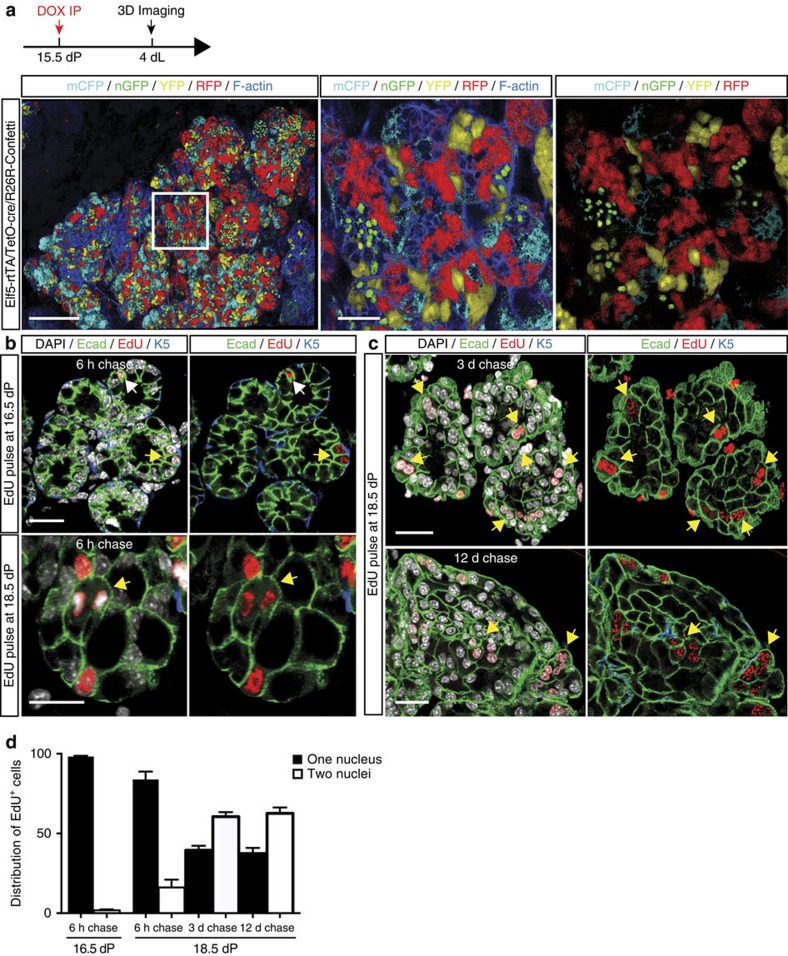
Binucleated cells are generated by failed cytokinesis and not by cell fusion. (**a**) Whole-mount 3D confocal image of a ductal portion from a representative Elf5-rtTA/TetO-cre/R26R-Confetti mouse. Elf5-rtTA/TetO-cre/R26R-Confetti mice were injected with doxycycline (DOX) at 15.5 days of pregnancy and analysed at 4 dL. Fusion was evaluated by determining whether cells express more than one colour. The whole-mount was labelled for F-actin (blue). Middle and right panels show optical sections from the selected region in the left panel. No multi-coloured cells were observed, indicating that cell fusion was not involved in the formation of binucleated cells (*n*=4 mice). Scale bars: 200 μm (whole-mount); and 50 μm (optical sections). (**b**) Optical section presented with (left) and without (right) DAPI staining from large whole-mounts of mammary glands from mice chased for 6 h after EdU injection at 16.5 dP (top) or 18.5 dP (bottom). Glands were stained for DAPI (white), EdU (red), Keratin 5 (blue) and E-cadherin (green). In the top panels, EdU^+^ cells contain only one nucleus; white arrow depicts a cell in S/G2 phase and the yellow arrow points to two cells that appear to have just undergone division. In the bottom panels, the yellow arrow depicts a binucleated cell. (**c**) Optical sections from whole-mounts of mammary glands isolated from mice injected with EdU at 18.5 dP and chased for 3 days (top) or 12 days (bottom). Yellow arrows depict binucleated cells that retain EdU. The whole-mounts were labelled for DAPI (white), E-cadherin (green), EdU (red) and K5 (blue). Scale bars: 20 μm (*n*=3 mice for each experiment). (**d**) Bar graph showing the percentage of EdU^+^ cells for luminal cells with either one or two nuclei chased for 6 h after EdU injection at 16.5 dP or chased for 6 h, 3 days or 12 days after EdU injection at 18.5 dP (*n*=3 mice per time-point). Error bars represent mean±s.e.m.

To explore potential mechanisms involved in the production of binucleated alveolar cells, we considered either cell fusion or failed cytokinesis. By exploiting the multi-colour confetti system^15^ combined with a doxycycline-inducible *cre* under the control of the *Elf5* promoter, we first examined whether a fusion mechanism might account for the formation of binucleated cells. *Elf5* is exclusively expressed in luminal progenitor cells in the virgin gland^8^ and the vast majority of luminal cells in the pregnant gland^16^. The confetti reporter locus was triggered in late pregnancy (15.5 days) in Elf5-rtTA/TetO-cre/R26R-Confetti mice to randomly activate the expression of one of its four colours, and scanned for the presence of multi-coloured cells at 4 days of lactation. No double-labelled cells were apparent, indicating that cell fusion had not occurred (Fig. 2a), such as occurs in the case of skeletal muscle cells.

We next turned to EdU-labelling studies to address cell division. Pregnant dams at 16.5 or 18.5 dP were injected with a single dose of EdU and subjected to 3D confocal imaging after a defined chase period. At 16.5 days, only dividing mononucleated cells were evident at 6 h after EdU injection. In contrast, at 18.5 days, binucleated EdU^+^ luminal cells appeared after a 6 h chase, with an abundance of these cells evident after analysis at 3 days (Fig. 2b). These data suggest that some alveolar cells can execute DNA replication in very late pregnancy but fail to undergo cytokinesis to produce two cells for lactation. Pulse-chase experiments in which mice were injected with EdU on day 18.5 of pregnancy and then analysed 3 and 12 days later showed that binucleated EdU^+^ cells are relatively long lived for highly metabolic cells (Fig. 2c). Analysis of the distribution of EdU^+^ cells in mono- versus bi-nucleated cells confirmed the shift towards binucleation at the onset of lactation (Fig. 2d). There are fewer EdU^+^ mononucleated cells in lactation compared with 18.5 days of pregnancy as these cells undergo one further round of division (with aborted cytokinesis) to generate cells comprising two nuclei.

### AURKA and PLK-1 are upregulated at the lactation switch

**Figure 3:**
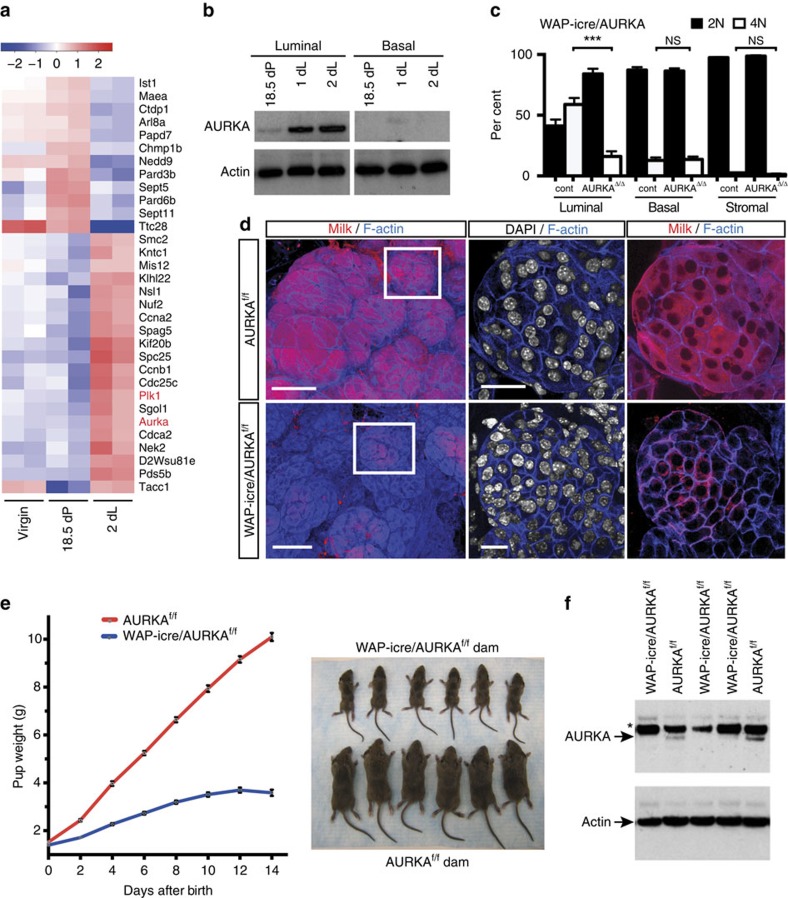
*AURKA* is induced at the onset of lactation and is essential for the formation of binucleated cells. (**a**) Heat map showing genes differentially expressed between the virgin state, late pregnancy and early lactation in sorted luminal cells for the cell division GO group. *AURKA* and *PLK1* are marked in red; *P*<0.05 for 2 dL versus 18.5 dP (using a TREAT-moderated *t*-test). (**b**) Representative western blot analysis (*n*=2) of AURKA expression in sorted luminal (Lin^−^CD29^lo^CD24^+^) and basal (Lin^−^CD29^hi^CD24^+^) cells isolated from pregnant (18.5 dP) or lactating glands (1 and 2 dL). (**c**) Bar graph of FACS data showing percentage of cells containing 2N or 4N DNA content in the luminal, basal and stromal cell compartments from WAP-icre/AURKA^f/f^ (AURKA^Δ/Δ^) and control AURKA^f/f^ mice at 2 dL. Error bars represent mean±s.e.m. (*n*=3 mice) ***P<0.001. (**d**) Representative whole-mount 3D confocal images of ductal portions and optical sections from the enlarged region for WAP-icre/AURKA^f/f^ glands (bottom) compared with control AURKA^f/f^ mammary glands (top). The whole-mounts were labelled for DAPI (white), milk (red) and F-actin (blue). Scale bars: 100 μm (whole-mounts); and 20 μm (optical sections). (**e**) Body weight of pups nursed by WAP-icre/AURKA^f/f^ dams versus littermate control AURKA^f/f^ dams (*n*=6). Error bars represent mean±s.e.m. *P*<0.0001. Representative photograph of pups nursed by a WAP-icre/AURKA^f/f^ dam versus an AURKA^f/f^ dam at 14 dL. (**f**) Western blot analysis (*n*=2) of expression of AURKA and Actin in WAP-icre/AURKA^f/f^ compared with AURKA^f/f^ glands at 2 dL. The asterix denotes a nonspecific band.

To interrogate possible genes that govern the balance between mononucleated and binucleated cells at the onset of lactation, we examined the RNA-seq expression profiles of genes differentially expressed between late pregnancy and early lactation in luminal cells^17^. *Aurora kinase A (AURKA)* emerged as one of the top hits upon analysis of upregulated genes in the cell cycle functional group (Fig. 3a). *AURKA* encodes a mitotic spindle-associated kinase that coordinates the functions of centrosomes, spindle formation and kinetochores required for mitotic progression and genomic integrity^18^. Quantitative PCR with reverse transcription (qRT–PCR) (Supplementary Fig. 5a) and western blot analysis (Fig. 3b) of sorted cells showed that AURKA was markedly upregulated in the luminal cell compartment at the onset of lactation relative to levels in late pregnancy. As anticipated, no change in AURKA protein levels was observed in the basal cell population. Evaluation of protein expression during lactation revealed that AURKA levels were high at the onset of lactation but declined to undetectable levels by day 4 (Supplementary Fig. 5b), suggesting that AURKA only functions around the lactation switch.

We next addressed the expression of other signalling kinases that are critical for cytokinesis, PLK-1 and AURKB^18^,^19^. PLK-1, a substrate of AURKA, was also markedly upregulated in early lactation relative to late pregnancy based on RNA-seq analysis (Fig. 3a), while no change was evident in AURKB expression, as confirmed by qRT–PCR analysis (Supplementary Fig. 5a). In summary, these data implicate the mitotic kinases AURKA and PLK-1 in the regulation of cytokinesis at the lactational switch.

Given that prolactin (Prl) is a key lactogenic hormone^20^,^21^, we explored whether Prl could induce AURKA expression and binucleation *in vivo* by treating mice for 3 days with Prl during late pregnancy (from 15.5 to 17.5 days), followed by analysis 6 h post-injection on day 17.5. Analysis of mammary glands showed a very low proportion of binucleated cells, implying that Prl alone is not sufficient to induce this process (Supplementary Fig. 6a,b). Since EGF is dramatically induced at the onset of lactation^17^, we next treated mice with a combination of Prl and EGF over the same time course. Strikingly, we observed the induction of binucleated cells (∼30%) concomitant with induction of AURKA by western blot analysis (Supplementary Fig. 6). Thus, Prl and EGF form part of the signal that instructs alveolar cells not to undergo cytokinesis in early lactation, thus leading to a binucleated cellular state.

### AURKA is essential for the formation of binucleated cells

To investigate whether AURKA plays a role in the formation of binucleated cells, we first conditionally deleted this gene using the WAP-icre strain that drives *cre* expression in differentiated alveolar cells from late pregnancy. Fluorescence-activated cell sorting (FACS) analysis and confocal imaging in 3D demonstrated that loss of *AURKA* profoundly disrupted the generation of binucleated cells at the onset of lactation. Only mononucleated cells were evident in 3D images of *AURKA-*deficient glands, with a greater than five-fold decrease in the number of cells with a 4N DNA content evident by FACS analysis (Fig. 3c,d and Supplementary Fig. 7a). Moreover, there was substantially less milk protein in these glands compared with that in control dams (Fig. 3d), and examination of pups nursed by WAP-icre/AURKA^f/f^ dams revealed severe stunting of pups compared with those nursed by AURKA^f/f^ or WAP-icre/AURKA^f/+^ dams (Fig. 3e). qRT–PCR analysis confirmed drastically lower levels of *α-casein, β-casein* and *WAP* gene expression in *AURKA*-deficient glands (Supplementary Fig. 7b), while western blotting validated deletion of *AURKA* (Fig. 3f). Importantly, EdU labelling at P0 after doxycycline-mediated deletion of AURKA showed the presence of EdU^+^ cells in AURKA-deficient glands (Supplementary Fig. 7c), indicating that cells could enter S phase and undergo DNA replication. This observation is compatible with a specific role for AURKA in the lactating mammary gland in the regulation of cytokinesis.

**Figure 4:**
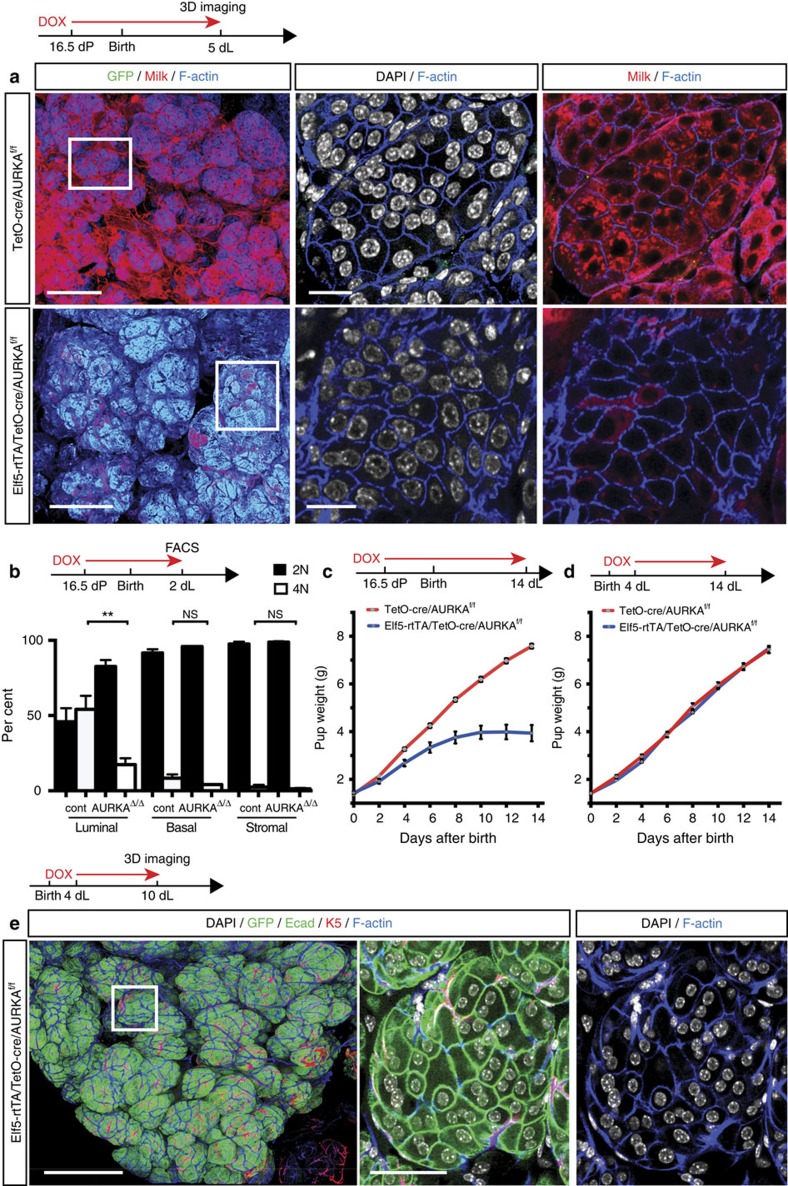
Temporal control of *AURKA* expression is important for the generation of binucleated alveolar cells. (**a**) Whole-mount 3D confocal images of ductal portions and optical sections from the enlargements for Elf5-rtTA/TetO-cre/AURKA^f/f^ gland (bottom) compared with control TetO-cre/AURKA^f/f^ mammary gland (top). The whole-mounts were labelled for DAPI (white), GFP (green), milk (red) and F-actin (blue). The animals were given DOX food from 16.5 dP until imaging analysis at 5 dL (*n*=4 mice). Scale bars: 100 μm (whole-mounts); and 20 μm (optical sections). (**b**) Bar graph of FACS data showing percentage of cells containing 2N or 4N DNA content in the luminal, basal and stromal cell compartments from Elf5-rtTA/TetO-cre/AURKA^f/f^ (AURKA^Δ/Δ^) and control AURKA^f/f^ mice at 2 dL, after DOX induction from 16.5 dP. Error bars represent mean±s.e.m. (*n*=3 mice). **P<0.01. (**c**) Body weight of pups nursed by Elf5-rtTA/TetO-cre/AURKA^f/f^ dams versus littermate control TetO-cre/AURKA^f/f^ dams. DOX food was given from 16.5 dP to 14 dL (six pups per dam; *n*= 3 dams). *P*<0.0001. (**d**) Body weight of pups nursed by Elf5-rtTA/TetO-cre/AURKA^f/f^ dams versus control dams. DOX was given from 4 to 14 dL (six pups per dam; *n*=2 dams). Error bars represent mean±s.e.m. (no significant difference). (**e**) Representative whole-mount 3D confocal image of a ductal portion from Elf5-rtTA/TetO-cre/AURKA^f/f^ mice. DOX food was given to mice from 4 dL until collection at 10 dL. The whole-mount was labelled for DAPI (white), E-cadherin/GFP (green), K5 (red) and F-actin (blue) to highlight the architecture of the gland (*n*=2 mice). Middle and right panels show optical sections from the selected area in the left panel. Most cells remain binucleated. Scale bars: 200 μm (whole-mount); and 30 μm (optical sections).

We next turned to a more sophisticated *cre* recombinase system to temporally delete the *AURKA* gene in late pregnancy versus early lactation utilizing a doxycycline-inducible *cre* under the control of the *Elf5* promoter^8^. Elf5 is a master transcriptional regulator of the secretory alveolar lineage^16^ and is highly expressed in luminal cells of pregnant and lactating mammary glands (Supplementary Fig. 8a). Evaluation of lactating mammary glands from Elf5-rtTA/TetO-cre/AURKA^f/f^ dams by 3D confocal imaging and FACS analysis showed a marked decrease in the proportion of binucleated cells relative to control Elf5-rtTA/AURKA^f/f^ dams at 2 days of lactation, following treatment with doxycycline from day 16.5 of pregnancy (Fig. 4a and Supplementary Fig. 8b). Notably, FACS analysis of control Elf5-rtTA/AURKA^f/f^ glands indicated that almost all binucleated cells (>98%) expressed *Elf5* (Supplementary Fig. 8b,c). *AURKA*-deficient glands also exhibited a dramatic decline in milk production, resulting in impaired lactation and the stunted growth of pups (Fig. 4a–c). For both the Elf5/TetO-cre and WAP-icre-driven models, 20–30% of pups in some litters were found to die around day 10 of lactation owing to insufficient milk production by *AURKA*-deficient mothers. A lower density of alveolar units persisted in these mammary glands on day 14 of lactation relative to both control and heterozygous glands (phenotypically normal) (Supplementary Fig. 9a–c). By contrast, doxycycline treatment from days 1 or 4 of lactation, when binucleated cells have already been generated, had no effect on lactogenesis or histology (Fig. 4d,e and Supplementary Fig. 9d). Induction of deletion from P0 resulted in a small decrease in lactogenic capacity and pup weight, although the histology of the glands appeared normal (Supplementary Fig. 9e).

### Inhibition of AURKA or PLK-1 activity prevents binucleation

**Figure 5:**
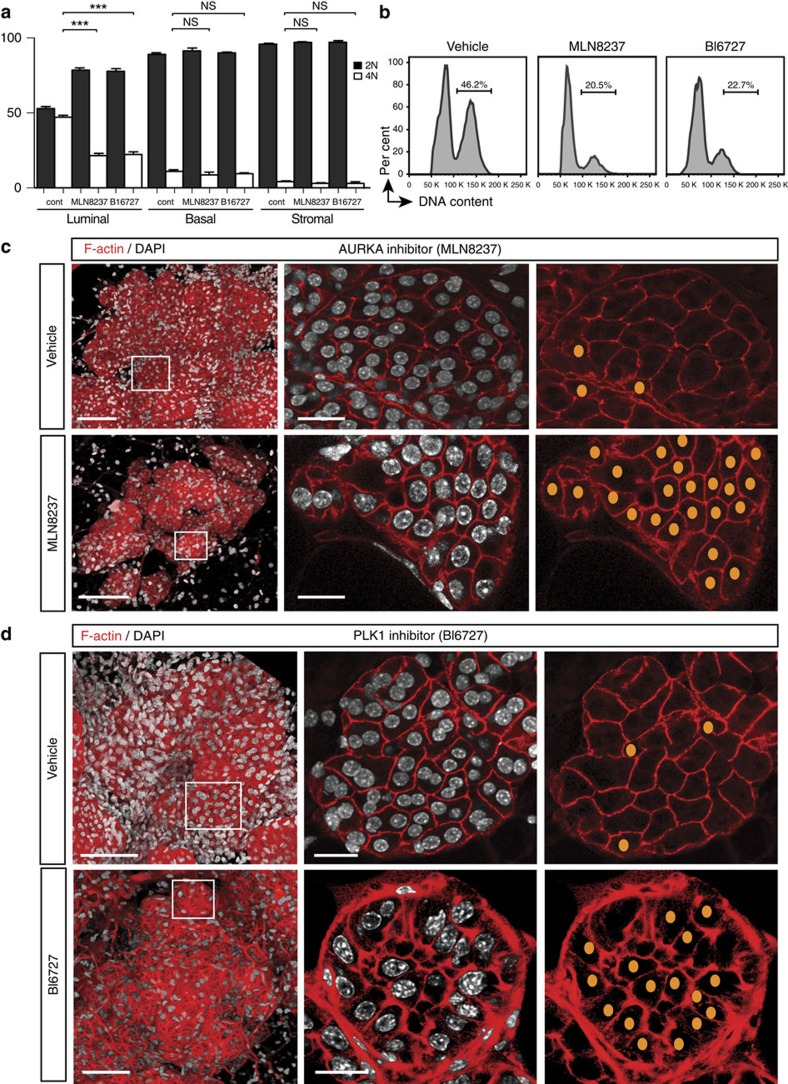
Pharmacological inhibition of AURKA or PLK-1 prevents the formation of binucleated cells. (**a**) Bar graph showing percentage of cells containing 2N or 4N DNA content in the luminal, basal and stromal cell compartments from mice treated from 16.5 dP with vehicle, MLN8237 (AURKA inhibitor) or BI6727 (PLK-1 inhibitor) and analysed by FACS at 2 dL. Error bars represent mean±s.e.m. (*n*=3 mice per treatment). ***P<0.001. (**b**) Representative FACS plots of DNA ploidy in the luminal populations from **a**. (**c**) Representative whole-mount 3D confocal images of ductal portions and optical sections from the enlarged regions for mice treated with vehicle or MLN8237. (**d**) Representative whole-mount 3D confocal images of ductal portions and optical sections from the enlarged regions for mice treated with vehicle or BI6727. In **c** and **d**, the whole-mounts were labelled for DAPI (white) and F-actin (red). In the right panels, nuclei in mononucleated cells within the middle panels are depicted schematically as orange dots. Scale bars: 100 μm (whole-mounts); and 20 μm (optical sections).

In addition to targeted deletion, we examined the effects of pharmacological inhibition of AURKA or PLK-1 on the formation of binucleated cells. Mice were treated with either the AURKA (MLN8237) or PLK-1 (BI6727) inhibitor, which directly inhibit kinase activity, or vehicle from 16.5 days of pregnancy and then mammary glands isolated from these mice were assessed at 2 days of lactation. FACS analysis showed a greater than twofold decrease in the proportion of alveolar cells with 4N DNA content by either inhibitor (Fig. 5a,b), while confocal 3D imaging revealed a predominance of mononucleated cells (Fig. 5c,d). No change was evident in the DNA ploidy of basal or stromal cells, thus serving as a control (Fig. 5a). These data also indicate that mitosis was not blocked upon inhibition of these mitotic kinases near the lactation switch, and thus does not account for the lack of binucleation. Furthermore, pups lacked milk in their stomachs, indicating that lactogenesis was impaired. These results are consistent with the notion that the increase in expression/activity of AURKA and PLK-1 occurring at the onset of lactation is critical for preventing cytokinesis and the formation of binucleated cells.

### Conservation of polyploid cells across mammalian species

**Figure 6:**
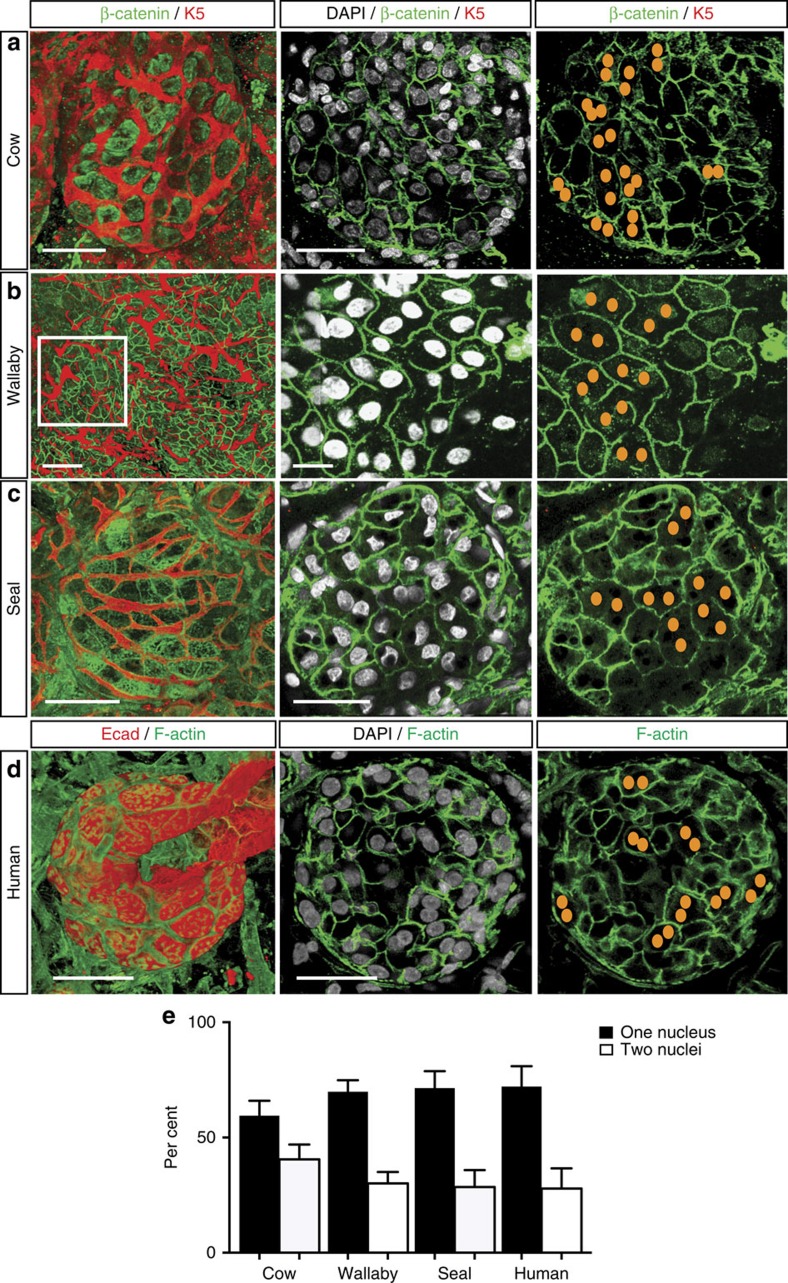
Presence of binucleated luminal cells in lactating mammary tissue from different mammalian species. (**a**) Whole-mount 3D confocal image of a ductal portion from paraffin-embedded (PE) mammary tissue from a late-pregnant cow (*n*=3). (**b**) Whole-mount 3D confocal image of a ductal portion from PE lactating mammary tissue from a wallaby (*n*=3). (**c**) Whole-mount 3D confocal image of a ductal portion from PE lactating mammary tissue from seal (*n*=3). (**d**) Whole-mount 3D confocal image of a ductal tree from fresh human lactating breast tissue (*n*=5). For each species, the depicted image is from the selected area shown in Supplementary Fig. 10a–d. The cow, seal and wallaby whole-mounts were labelled for Keratin 5 (red), β-catenin (green) and DAPI (white), whereas human tissue was stained for E-cadherin (red), F-actin (green) and DAPI (white). In the right panels, nuclei in binucleated cells within the middle panels are depicted schematically as orange dots. Scale bars: 20 μm (whole-mounts); and 10 μm (optical sections). (**e**) Bar graph showing per cent of binucleated and mononucleated cells in alveoli (*n*=3–5 samples). More than 20 alveoli (>1,000 cells) were counted for each species. Error bars represent mean±s.e.m.

Given that polyploidy is essential for maximal lactogenesis in the mouse, we speculated that it might be a feature of other mammalian species. To this end, we examined lactating tissue from human breast, seal and wallaby, and of late pregnant tissue from cow. As binucleated cells are not readily detectable in two-dimensional sections, we performed 3D confocal microscopy on thick tissue sections (up to 400 μm) from paraffin or OCT blocks using a modification of the procedure for whole-mounted fresh tissue. Fresh human tissue was also assessed, yielding similar findings. Remarkably, binucleated cells were prominent in all species examined, with ∼30% observed in human, seal and wallaby tissue, and 40% in bovine mammary tissue (Fig. 6a–e and Supplementary Fig. 10).

## Discussion

Through the use of 3D confocal imaging, our studies have uncovered the presence of remarkable numbers of polyploid alveolar cells in the lactating mouse mammary gland. These cells arise through a blockade in cytokinesis and have a considerably larger volume than cells comprising a single nucleus. Moreover, they play an essential role in lactogenesis. During lactation, the sole function of alveolar epithelial cells is to generate large amounts of milk protein, lipid and carbohydrate, to support the offspring^6^. Polyploid cells presumably evolved around the onset of lactation to enhance milk production. The larger cytoplasmic volume of binucleated alveolar cells likely accommodates more ribosomes, ER and Golgi required for the biosynthesis of milk protein and lipid^22^. In addition, these larger cells have a greater apical surface area to enable more efficient milk secretion.

Mechanistically, the mitotic kinases AURKA and PLK-1 have emerged as key regulators of cytokinesis of mammary alveolar cells at the lactogenic switch. The function of AURKA is not only pivotal to centrosome function and mitotic assembly^18^,^19^, but there is emerging evidence to suggest that it is implicated in the early stages of cytokinesis^23^,^24^,^25^. The two lactation-specific *AURKA* knockout models explored here highlight the importance of binucleated alveolar cells for efficient milk production and lactation. Only deletion of *AURKA* at the switch to lactation inhibited the formation of binucleated cells and milk synthesis. These findings underscore the importance of timing of expression of this serine/threonine kinase. Moreover, the precise level of AURKA expression has been shown to be crucial for proper cytokinesis, where inhibition^26^ or overexpression^27^,^28^,^29^ of this gene resulted in failed cytokinesis and the formation of binucleated cells. Pertinently, AURKA is frequently amplified in breast cancer, and overexpression of this gene in mouse genetic models accelerates oncogenesis by inducing genetic instability and the formation of binucleated cells, which succumb to apoptosis^29^,^30^. In a physiological setting, our findings uncover an important role for AURKA in the generation of polyploid milk-producing cells at the switch to lactation.

Inhibition of PLK-1, a substrate of AURKA and an important regulator of cytokinesis^19^, also led to a block in cytokinesis at the lactational switch. These data recapitulate those seen upon genetic ablation or pharmacological inhibition of AURKA, suggesting that control of cytokinesis in lactation is a shared function of these kinases. Overexpression of PLK-1 has been previously reported to cause multinucleation and interfere with cytokinesis^28^, emphasizing the importance of the absolute levels of mitotic kinases in control of the cell cycle. Together, these data suggest that augmented expression (and activity) of these two kinases, specifically at the onset of lactation, is key to the prevention of cytokinesis after chromosome segregation and nuclear division. In the mammary gland, moderate levels of AURKA and PLK-1 are presumably required for mitotic progression, whereas increased levels of these kinases at a specific temporal window trigger a failure of cytokinesis to enable the production of binucleated alveolar cells in lactation.

Polyploidy has been demonstrated to be a feature of specific mammalian cell types including the megakaryocyte, hepatocyte, trophoblast giant cell and cardiomyocyte^31^,^32^. These cells differ in their function and number of chromosome sets, and although it is presumed that polyploidy confers a considerable advantage on tissue growth or differentiation, its functional significance is poorly understood. There is evidence to suggest that polyploidy may be important for cardiac muscle under stress conditions, since mice lacking a gene necessary for polyploidy were normal at baseline but their hearts pumped substantially less blood after an infarct^33^. Similarly, the high proportion of polyploid cells in the liver may be required to mediate a proper stress-response^34^. In the mammary gland, which evolved as an epidermal appendage over 300 million years ago, we demonstrate a crucial role for polyploidy in enabling effective lactation. Our data further suggest that polyploid alveolar cells evolved as a conserved feature in lactation to support the nutritional requirements of the young across all mammalian species.

## Methods

### Mice

AURKA^f/f^ mice^35^ were obtained from the Jackson Laboratory. WAP-icre^36^, TetO-cre^37^ and R26R-Confetti mice^15^ were kind gifts from G. Schuetz (German Cancer Research Centre, Heidelberg), K. Schönig (Central Institute of Mental Health, Mannheim) and H. Clevers (Hubrecht Institute, Utrecht), respectively. Elf5-rtTA-GFP mice were generated at the Walter and Eliza Hall Institute of Medical Research (WEHI)^8^. FVB/N mice were provided by the animal facility of WEHI. All mice were bred and maintained in our animal facility according to the institutional guidelines, and all experiments were approved by the WEHI Animal Ethics Committee.

For timed pregnancies, adult female mice were mated with FVB/N stud males, scored by the presence of a vaginal plug and confirmed by the examination of embryos at the time of mammary gland collection. For lactation experiments, adult female mice were mated with FVB/N stud males and six offspring per litter were maintained. For *in vivo* EdU (5-ethynyl-2-deoxyuridine; Invitrogen) label, mice were injected intraperitoneally (IP) with 0.2 mg of EdU (200 μl, 1 mg ml^−1^ in PBS). For induction of deletion of *AURKA* in the Elf5-rtTA/TetO-cre/AURKA^f/f^ line, mice were administered doxycycline via food pellets^8^ from 16.5 days of pregnancy (dP), or 0 (birth), 1 or 4 days lactation (dL) until collection of mammary glands for analysis to ensure efficient deletion. For induction of expression of the Confetti reporter in the Elf5-rtTA/TetO-cre/R26R-Confetti^KI/+^ line, mice were injected IP (once) with 2 mg of doxycycline (100 μl, 20 mg ml^−1^ in PBS; Sigma) at 15.5 days of pregnancy. To inhibit the kinase activity of AURKA and PLK-1 kinase, MLN8237 (1.25 mg per mouse, daily) or BI6727 (0.75 mg per mouse, daily) were administered, respectively, via oral gavage to pregnant mothers from 16.5 dP to 1dL and mice were collected and analysed at 2 days of lactation. To test the effect of prolactin/EGF on the generation of binucleated cells, pregnant mothers were subcutaneously injected with prolactin (0.2 mg per mouse, twice per day) with or without IP injection of EGF (0.01 mg per mouse, twice per day) between 15.5 and 17.5 dP. Mice were then collected and analysed 6 h after the last injection.

### Human breast tissue

Human breast tissue was obtained from consenting individuals through the Victorian Cancer Biobank with approval from the Human Research Ethics Committee of The Walter and Eliza Hall Institute of Medical Research. Fresh samples were normal breast tissue (confirmed by pathology) from reduction mammoplasties. Three paraffin-embedded (PE) and two fresh samples were analysed.

### Mammary cell suspension preparation and flow cytometry

Mammary glands were dissected from adult female mice, and single-cell suspensions were prepared^14^. To assess ploidy through measuring DNA content, cells were first incubated with 20 μg ml^−1^ Hoechst 33342 (Invitrogen) at 37 °C shaker for 1 h and maintained in a solution containing 20 μg ml^−1^ Hoechst 33342 in all steps after staining to prevent the release of dye from the cells. Otherwise, cells were directly stained with antibodies on ice for 25 min. The following antibodies were used: PE anti-mouse CD24 (rat, clone M1/69, BD; 1:200 dilution), APC/cy7 anti-mouse/rat CD29 (rat, clone HMb1-1, BD; 1:200 dilution), APC anti-mouse CD45 (rat, clone 30-F11, BD; 1:100 dilution), APC anti-mouse Ter119 (rat, clone Ter-119, BD; 1:100 dilution) and APC anti-mouse CD31 (rat, clone MEC13.3, BD; 1:50 dilution). Cells were re-suspended in 4 μg ml^−1^ 7-AAD (Sigma) before analysis to exclude dead cells. FACS analysis and sorting were performed using a FACS LSRIIC and an Aria (Becton Dickinson), respectively. The Lin^–^ population was defined as Ter119^–^CD31^–^CD45^–^ (ref. 14). FACS data were analysed using FlowJo software (Tree Star).

### Whole mounting and histology of mammary glands

For whole-mount analysis, mammary glands were fixed in Carnoy's solution containing ethanol, chloroform and glacial acetic acid (6:3:1) at room temperature for 48 h before staining with carmine alum. Mouse mammary glands were fixed in 4% paraformaldehyde, embedded in paraffin, sectioned and then stained with haematoxylin and eosin.

### Western blot analysis

FACS-sorted cells were directly lysed in RIPA buffer containing 1 × complete mini protease inhibitor cocktail (Roche) and 1 × Roche PhosSTOP phosphatase inhibitor cocktail (Roche). Whole mammary gland lysates were prepared by grinding tissue in liquid nitrogen and solubilizing in the RIPA buffer. The following primary antibodies were used for western blot analysis: anti-AURKA (mouse, clone 4/IAK1, BD; 1:500 dilution) and anti-beta-Actin (mouse, clone AC-15, Sigma; 1:5,000 dilution). All uncropped Western blots can be found in Supplementary Fig. 11.

### RNA preparation and quantitative RT–PCR analysis

Total RNA was prepared using the RNeasy Micro kit (Qiagen) from FACS-sorted luminal cells isolated from the mammary glands of FVB/N dams at 18.5 dP or 2 days of lactation, or from total mammary tissue derived from *AURKA-*deficient or control mice at 14 days of lactation. Reverse transcription was carried out using oligo(dT) primer and SuperscriptIII reverse transcriptase (Invitrogen, MA, USA). Quantitative RT–PCR analysis was performed using a Rotorgene RG-6000 (Corbett Research, Australia) and normalized against 18S ribosomal RNA.

### Whole-mount preparation of fresh tissue for 3D confocal imaging

Tissues were fixed in 4% paraformaldehyde and incubated overnight at 4 °C with primary antibodies. The following day, tissues were incubated with secondary antibodies, DAPI and phalloidin. Tissues were subsequently incubated in 80% glycerol before dissection for 3D imaging, as previously described^8^. The following primary antibodies were used for 3D confocal imaging: anti-E-cadherin (rat, clone ECCD-2, Invitrogen; 1:200 dilution), anti-Keratin 5 (rabbit, Covance; 1:500), anti-β-catenin (mouse, Cell Signaling; 1:500), anti-Milk (Accurate Chemical and Scientific Corporation; 1:1,000), anti-giantin (rabbit, Abcam; 1:250), cleaved caspase-3 (rabbit, clone D3E9, Cell Signaling; 1:100 dilution) and anti-GFP (chicken, Abcam; 1:500). Secondary antibody: Alexa Fluor (Invitrogen; 1:500). F-actin was stained with Alexa Fluor 647 Phalloidin (Invitrogen; 1:50 dilution). DAPI (Invitrogen) was used at 1 μg ml^−1^. EdU labelling was performed using the Click-it kit (Invitrogen).

### Preparation of embedded mammary tissue for confocal microscopy

Collection of mammary tissue from the cow, tammar wallaby (*Macropus eugenii*) and cape fur seal (*Arctocephalus pusillus pusillus*) is described elsewhere^38^,^39^,^40^. Three lactating mammary tissues embedded in OCT (Optimal Cutting Temperature) were analysed for seal; one paraffin-embedded (PE) and two OCT-embedded lactating samples were analysed for wallaby; three PE bovine specimens from late pregnancy were analysed. Human tissue was either fresh or PE (*n*=5). For 3D imaging, we prepared thick sections (up to 400 μm) using a microtome (Leica RM2) and then dewaxed using conventional procedures^41^. An antigen retrieval step was then performed in DAKO Target Retrieval pH9 solution at 90 °C for 30 min. OCT-embedded floating sections were cut using a cryostat (Microm HM550, Thermo Scientific) and then fixed in 4% paraformaldehyde for 30 min before immunostaining according to Rios *et al.*^8^.

### Imaging quantification for cell volume analysis

We performed cell volume measurements using the surface module in Imaris and binary masks produced from a segmentation process developed in FIJI. The segmentation method involved filtering, thresholding and inverting the [MEMBRANE STAIN/CHANNEL] and running a watershed algorithm seeded with points furthest from all membranes. The resulting particles were filtered for size to remove any clearly incorrect regions. The segmentation was manually validated on a slice-by-slice basis via a user-interface allowing selection of individual cells, where the segmentation was consistent with the cell morphology and staining. Any missing slices (or slices that were clearly incorrect) were filled in by interpolating between the two nearest slices assessed to be accurate. Once cell selection and validation had been performed, a binary mask of only the cells of interest was imported into Imaris for the volume measurements. Approximately 50 cells were analysed per mouse (*n*=3) at each time-point.

### Imaging quantification of nuclei

The module cell counter from Image J was used to count the number of cells with one or two nuclei in 3D images.

## Additional information

**How to cite this article:** Rios, A. C. *et al.* Essential role for a novel population of binucleated mammary epithelial cells in lactation. *Nat. Commun.* 7:11400 doi: 10.1038/ncomms11400 (2016).

## Supplementary Material

Supplementary Information Supplementary Figures 1-11

Supplementary Movie 1 Animation made with the Imaris software showing a three dimensional reconstruction of a ductal tree portion (volume 1.8 mm^3^) from a FVB/N mouse at 2 days of lactation. The whole-mount was labelled with DAPI (white), E-cadherin (green) and F-actin (red).
