# Supplementary material for: Essential role for a novel population of binucleated mammary epithelial cells in lactation
Source: Nat Commun. 2016 Apr 22;7:11400. doi: 10.1038/ncomms11400 (PMC4844753; doi:10.1038/ncomms11400)
Supplement: Supplementary Information — Supplementary Figures 1-11 [file ncomms11400-s1.pdf]

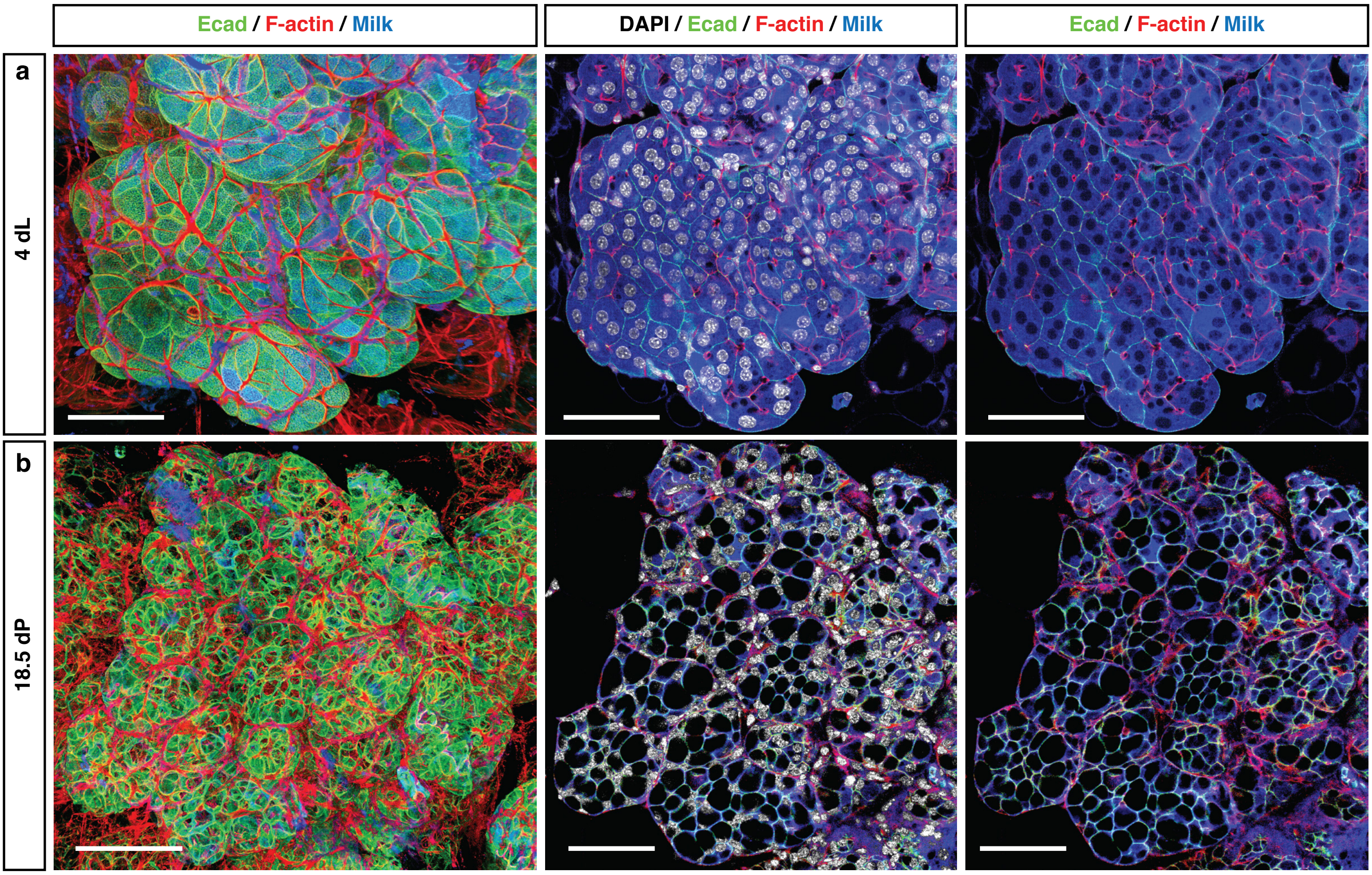

Supplementary Figure 1. Milk protein expression in late pregnant and early lactating mammary glands
(**a**) Representative whole-mount 3D confocal image of a mammary ductal portion from a FVB/N mouse at 4 days of lactation (4 dL). The gland was stained for DAPI (white), E-cadherin (green), F-actin (red), and Milk (blue). Middle and right panels show optical sections of the left panel. (**b**) Representative whole-mount 3D confocal image of a mammary ductal portion from a FVB/N mouse at 18.5 days of pregnancy (18.5 dP). The gland was immunolabelled as in **a**. Middle and right panels show optical sections of left panel. *n* = 3 mice for each time-point. Scale bars: 80 μm (whole-mounts); and 50 μm (optical sections).

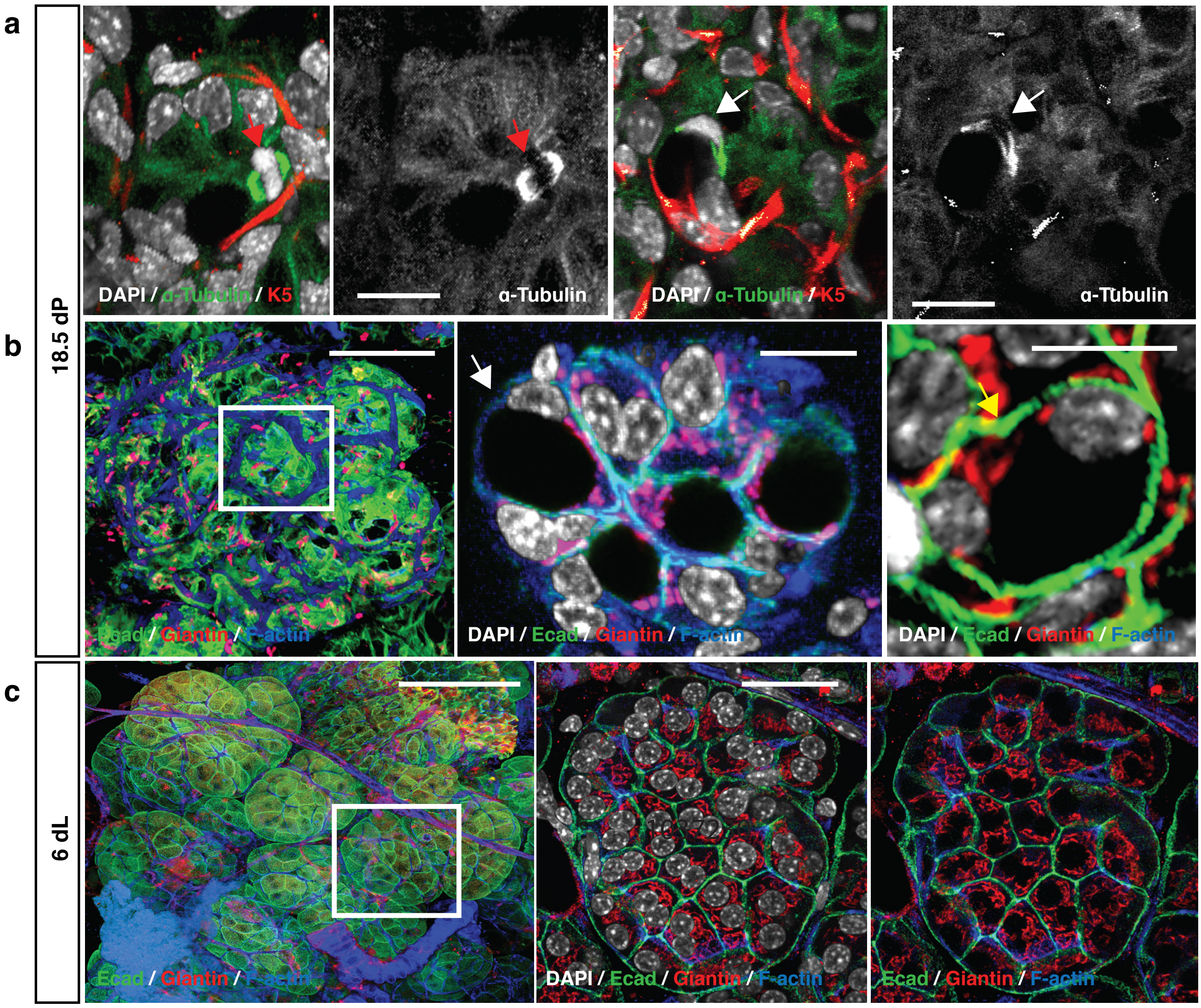

Supplementary Figure 2. Analysis of distribution of mitotic spindle and Golgi in mammary alveolar luminal cells in late pregnancy and early lactation

(**a**) Optical sections from a whole-mount 3D confocal image of a ductal portion stained with DAPI (white), α-tubulin (green) and Keratin 5 (red). In many cells, the mitotic spindle was found to be aberrantly located on the periphery of the cell, as depicted by the white arrow (right panels), in contrast to the symmetrical alignment observed in normal diploid cells (red arrow in left panels) (n = 4 mice). Scale bars: 10 μm. (**b**) Whole-mount 3D confocal image of a ductal portion from a FVB/N mouse at 18.5 dP and optical sections from the enlarged region. The whole-mount was stained with DAPI (white), E-cadherin (green), Giantin (red) and F-actin (blue). The organelles and nuclei are located on the periphery of the cell due to the presence of lipid droplets, as shown in the cell with one nucleus (middle panel, white arrow) and another with two nuclei (right panel, yellow arrow) (n = 3 mice). Scale bars: 30 μm (whole-mount); and 10 μm (optical sections). (**c**) Whole-mount 3D confocal image of a ductal portion of a mammary gland at 6 dL and optical sections stained with DAPI (white), E-cadherin (green), Giantin (red) and F-actin (blue) (n = 3 mice). Scale bars: 50 μm (whole-mount); and 20 μm (optical sections).

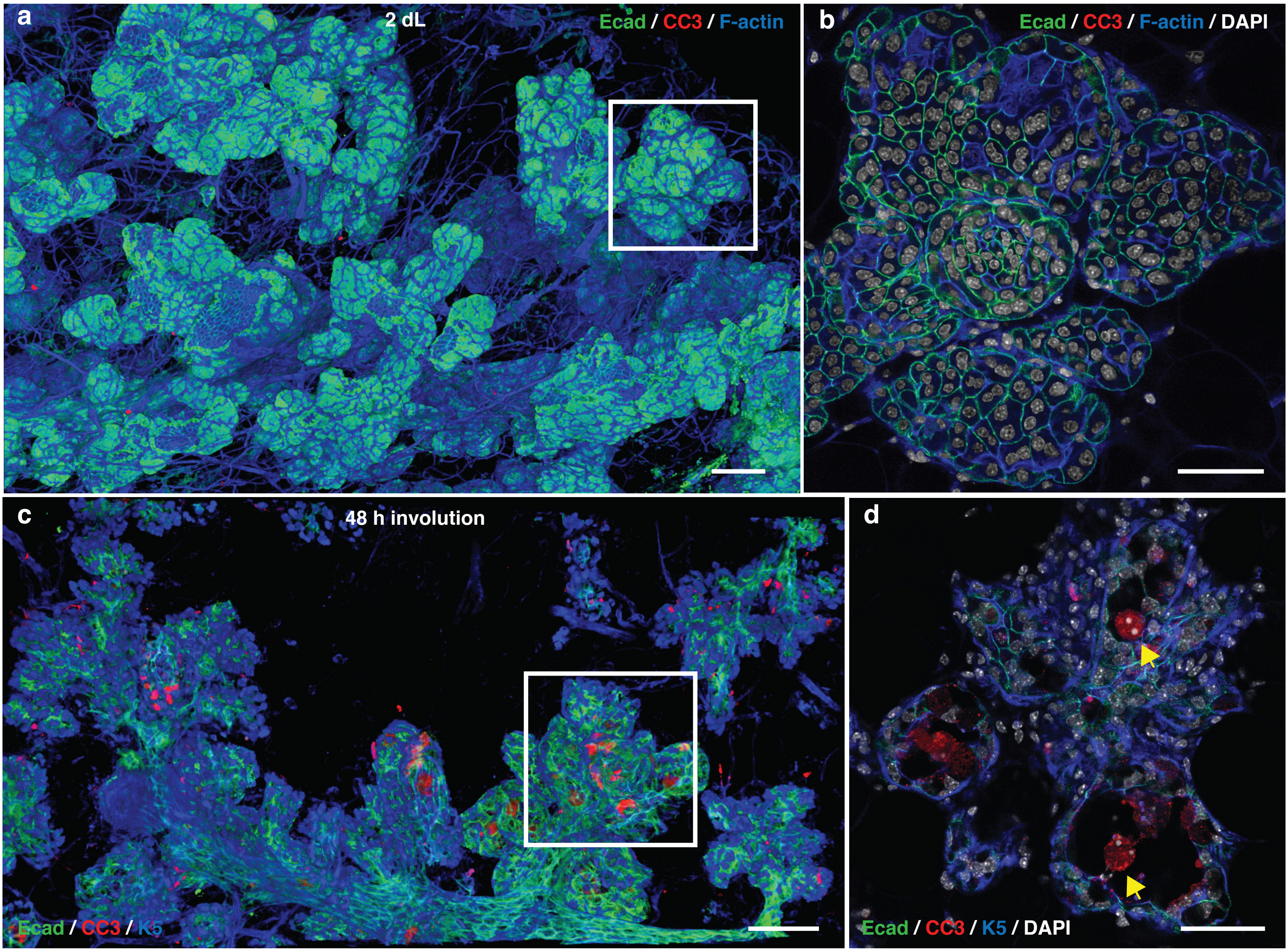

Supplementary Figure 3. Binucleated cells are prone to programmed cell death in early involution
(**a**) Whole-mount 3D confocal image of a ductal portion from a FVB/N dam at 2 days lactation stained with DAPI (white), E-cadherin (green), cleaved caspase-3 (CC3, red) and F-actin (blue). (**b**) Optical section from the enlargement in **a** showing a paucity of CC3$^+$ cells (*n* = 3 mice). (**c**) Whole-mount 3D confocal image of a ductal portion from a FVB/N dam in early involution (48 hours) stained with DAPI (white), E-cadherin (green), CC3 (red) and Keratin 5 (blue). (**d**) Optical section from the enlargement in **c**: the yellow arrows depict dying (CC3$^+$) binucleated cells in the lumen of the alveoli (*n* = 3 mice). Scale bars: 100 μm (whole-mounts); and 50 μm (optical sections).

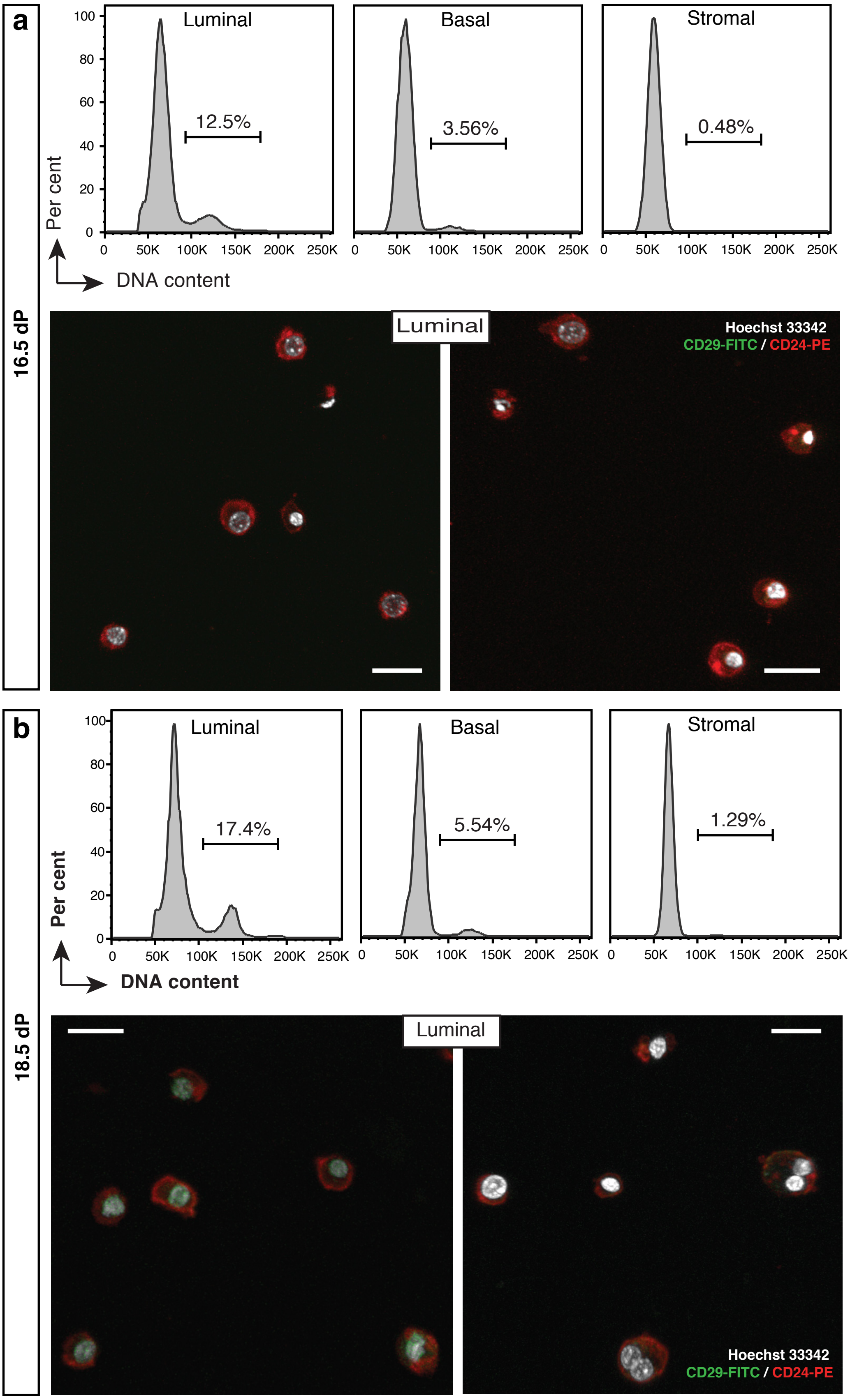

Supplementary Figure 4. Cell cycle and imaging analyses reveal the presence of binucleated cells from 18.5 days of pregnancy

(**a**) Representative DNA ploidy FACS plots of the luminal, basal and stromal populations from mammary glands at 16.5 days pregnancy (dP), showing the percentage of cells that contain 4N DNA content in each population. Confocal images showing sorted cells from the luminal population with either 2N (left panel) or 4N DNA content (right panel). (**b**) Representative DNA ploidy FACS plots of luminal, basal and stromal populations from an 18.5 dP mouse, and confocal images of sorted cells from the luminal population containing either a 2N (left panel) or 4N DNA content (right panel). Scale bars: 20 μm (*n* = 3 mice per time-point).

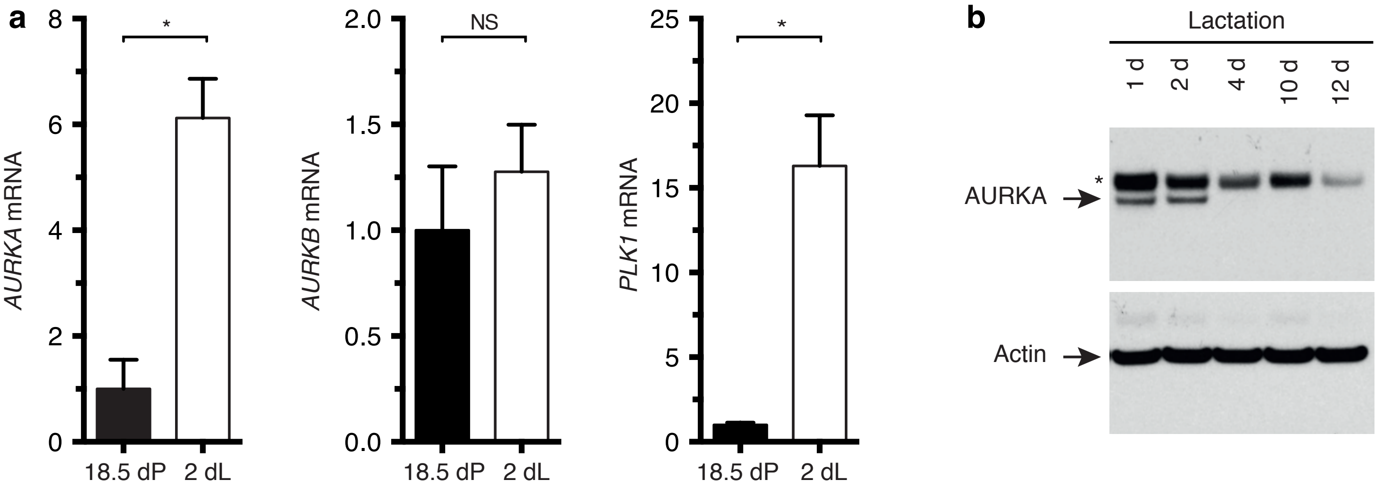

Supplementary Figure 5. Expression of mitotic kinases at the lactation switch
(**a**) qRT-PCR analysis of the relative expression of AURKA, AURKB and PLK-1 mRNA in FACS-sorted luminal cells from FVB/N mothers at 18.5 days of pregnancy or 2 days of lactation (n = 3). Error bars represent mean ± s.e.m. *$p<0.05$. (**b**) Western blot analysis of total mammary gland lysates at different time-points of lactation for AURKA and Actin expression (n = 2). *denotes non-specific band.

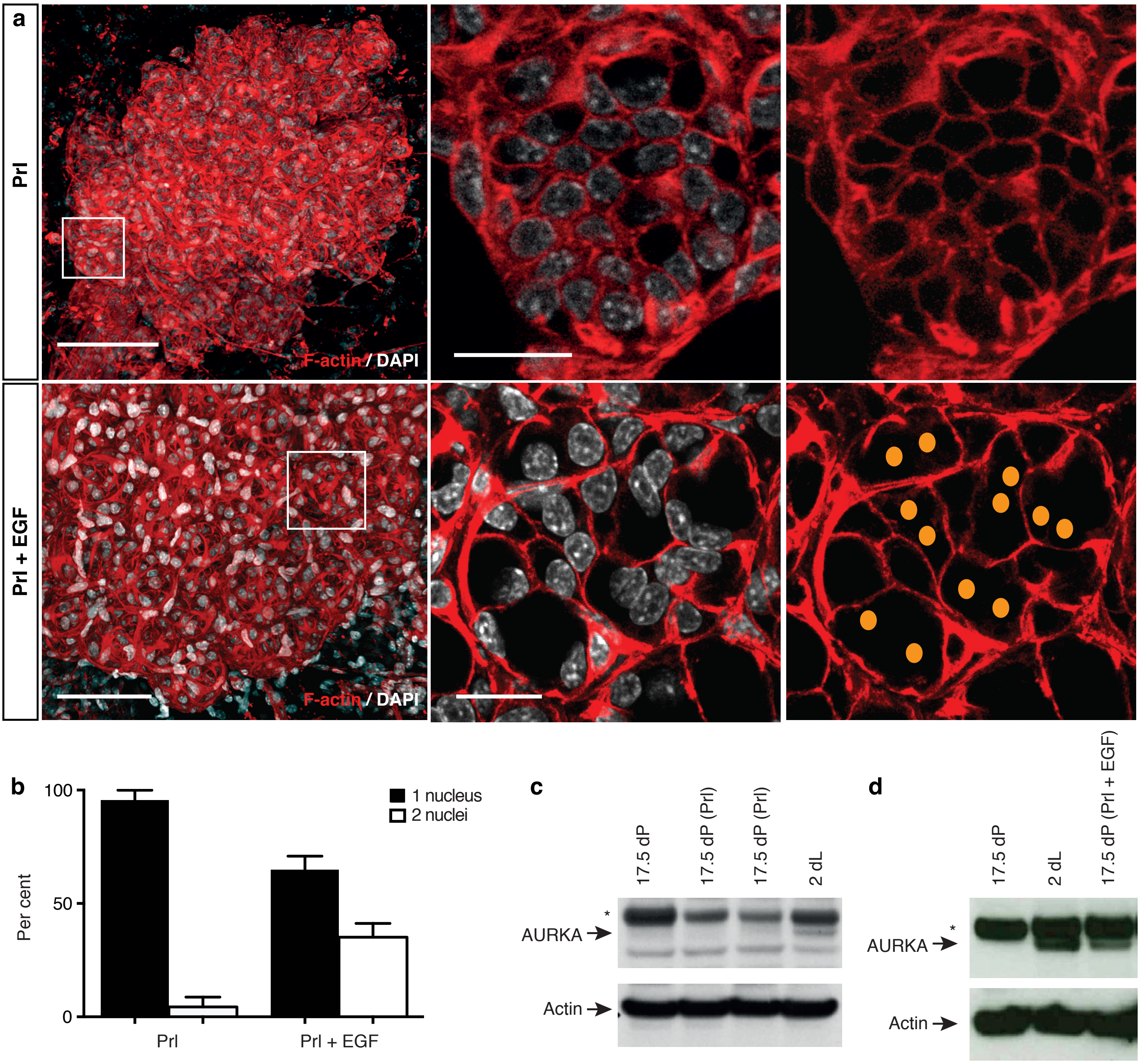

Supplementary Figure 6. Prolactin and EGF can induce binucleation of mammary alveolar cells *in vivo*
(**a**) Whole-mount 3D confocal images of a mammary ductal portion from FVB/N dams treated with prolactin (Prl) alone or Prl plus EGF from 15.5 days of pregnancy (dP) and collected at 17.5 dP. Glands were stained with DAPI (white) and F-actin (red). Optical sections from enlargements are shown. Nuclei in binucleated cells within the middle panels are depicted schematically as orange dots. Scale bars: 100 μm (whole-mounts); and 20 μm (optical sections). (**b**) Bar graph showing the percentage of binucleated and mononucleated cells in the mammary alveoli of treated mice. More than 20 alveoli (>1000 cells) were counted for each treatment. Error bars represent mean ± s.e.m. (**c**, **d**) Western blots showing AURKA and Actin expression in total mammary gland lysates from FVB/N dams treated with prolactin alone (Prl) or Prl plus EGF from 15.5 dP and collected at 17.5 dP. *denotes non-specific band (n = 4 mice for Prl; n = 2 for Prl plus EGF).

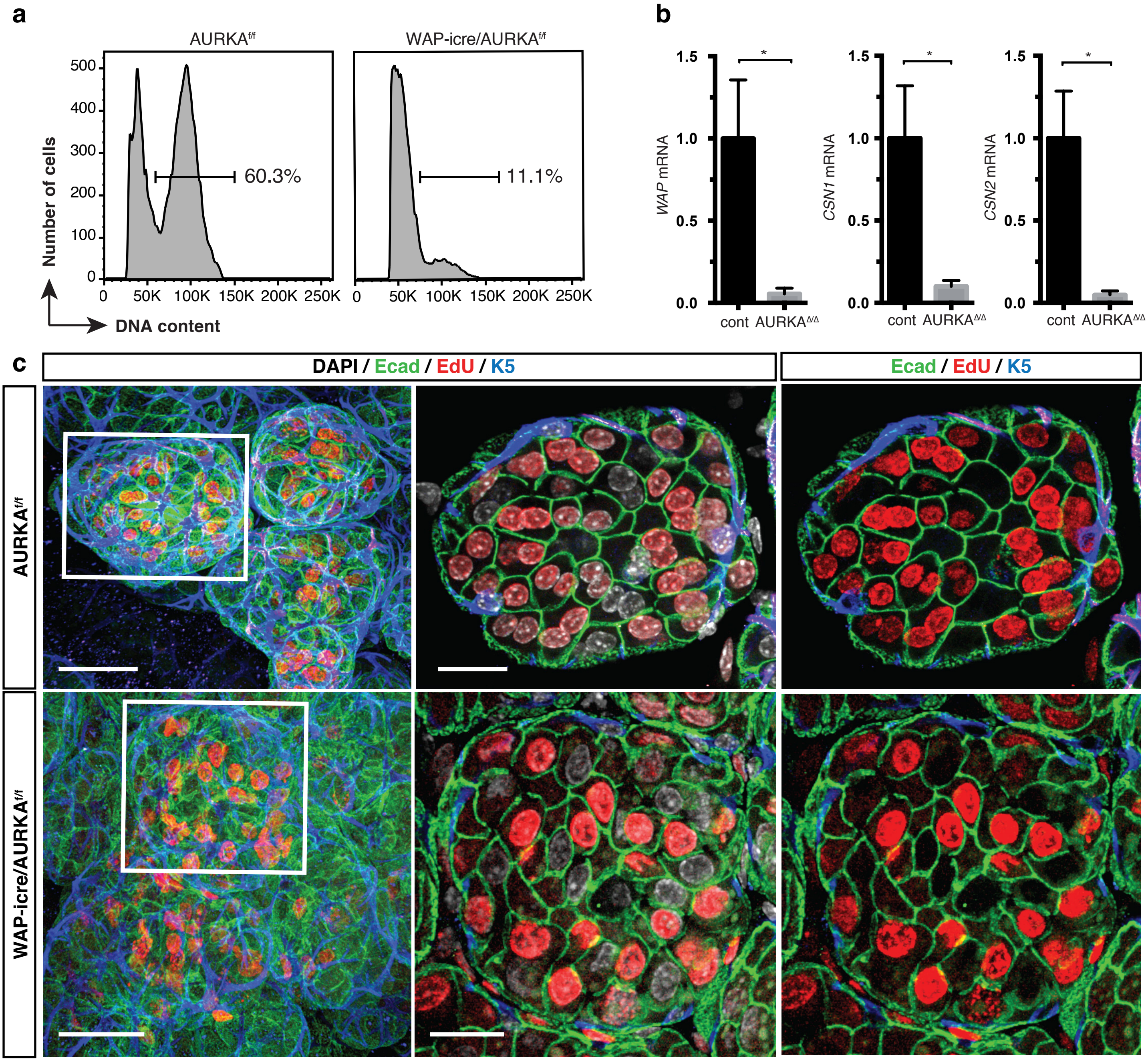

Supplementary Figure 7. AURKA-deficient cells are not blocked in mitosis

(**a**) Representative DNA ploidy FACS plots for the luminal population of mammary glands from WAP-icre/AURKA$^{f/f}$ and control AURKA$^{f/f}$ dams at 2 days of lactation (*n* = 3 mice). (**b**) qRT-PCR analysis of expression of milk genes WAP, CSN1 and CSN2 in WAP-icre/AURKA$^{f/f}$ (AURKA$^{\Delta/\Delta}$) and control AURKA$^{f/f}$ (cont) mammary glands (*n* = 3 mice per genotype). Error bars represent mean ± s.e.m. *p<0.05. (**c**) Representative whole-mount 3D confocal images of a ductal portion from control AURKA$^{f/f}$ or WAP-icre/AURKA$^{f/f}$ dams injected with EdU (0.2 mg/mouse, twice/day) from birth to collection at 2 days of lactation (*n* = 2 mice). Glands were stained with DAPI (white), E-cadherin (green), EdU (red) and Keratin 5 (blue). Optical sections from enlargements are shown. Scale bars: 50 μm (whole-mounts); and 20 μm (optical sections).

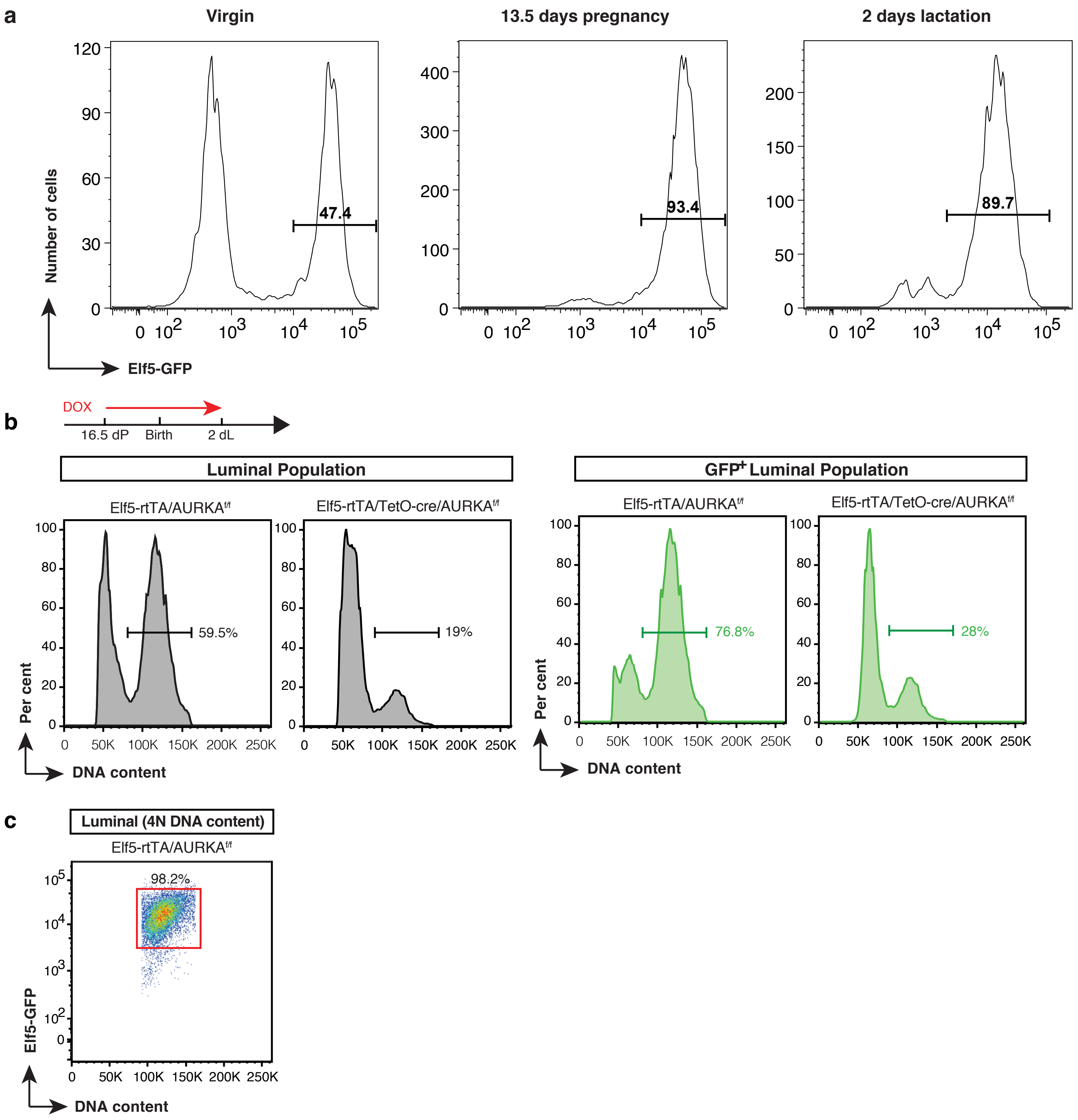

Supplementary Figure 8. Elf5-driven AURKA-deficient mammary glands lack binucleated cells in lactation
(**a**) Representative FACS plots showing the percentage of cells in mammary glands from virgin, mid-pregnant and early lactating Elf5-rtTA-GFP+ mice (n = 3). (**b**) Representative FACS plots showing DNA ploidy of total luminal (left panels) and Elf5-GFP+ luminal cells (right panels) from Elf5-rtTA/TetO-cre/AURKA$^{f/f}$ and control mammary glands at 2 dL. DOX food was administered from 16.5 dP (n = 2 mice). (**c**) Representative FACS plots showing that almost all binucleated cells are Elf5-GFP+ following analysis of luminal cells from the mammary glands of Elf5-rtTA/AURKA$^{f/f}$ control dams at 2 dL.

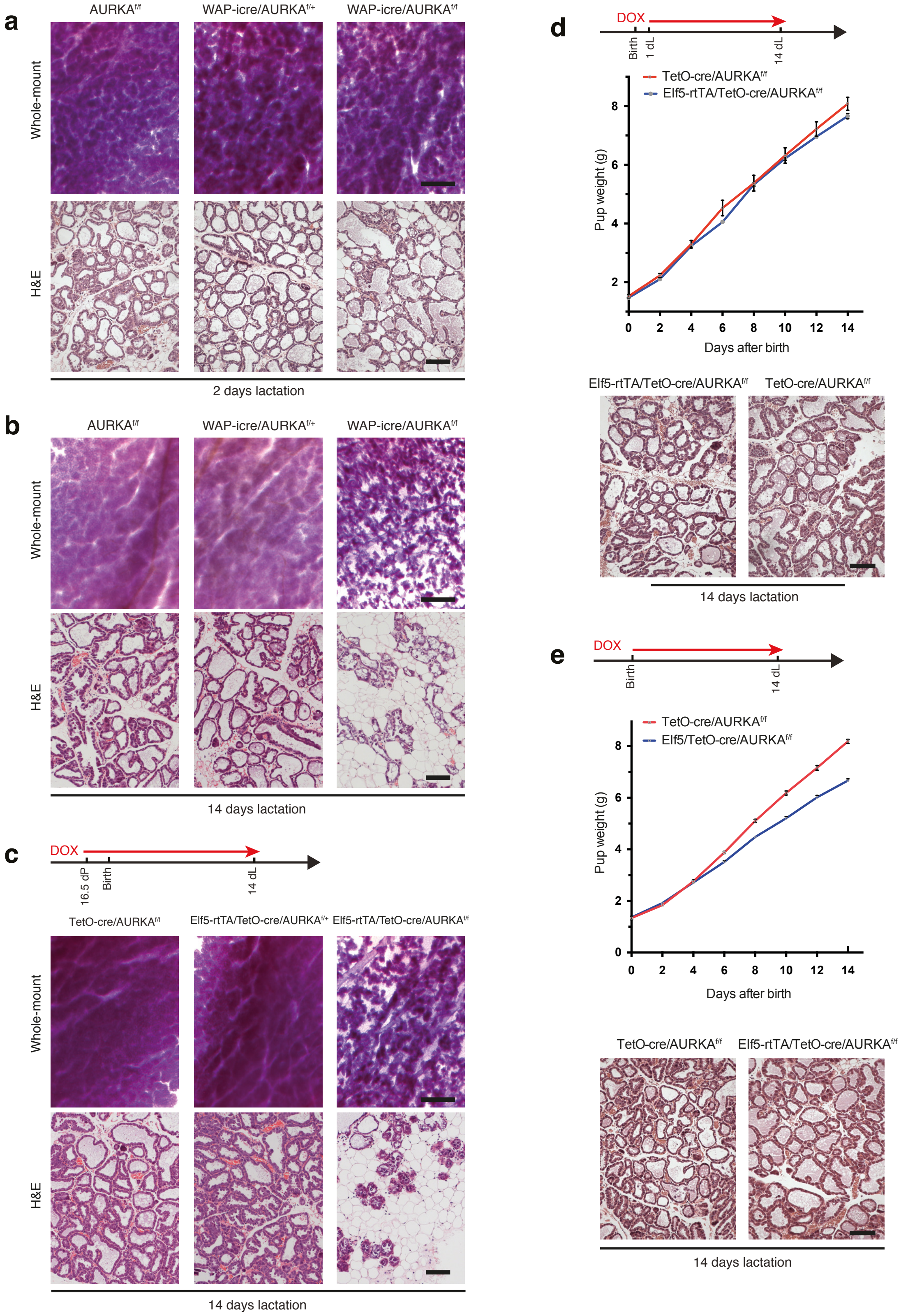

Supplementary Figure 9. Histological analysis of AURKA-deficient mammary glands and lack of phenotype in the Elf5-driven model induced after parturition

(**a**, **b**) Representative whole-mount and H&E sections of mammary glands from WAP-icre/AURKA$^{f/f}$, heterozygous and control dams at 2 days of lactation (dL) (**a**) and 14 dL (**b**) ($n = 6$ mice per genotype per time-point). (**c**) Representative whole-mount and H&E sections of mammary glands from Elf5-rtTA/TetO-cre/AURKA$^{f/f}$, heterozygous and control dams at 14 dL ($n = 3$ mice per genotype). Mice were given DOX food from 16.5 dP to 14 dL. (**d**, **e**) Graphs showing body weight of pups nursed by Elf5-rtTA/TetO-cre/AURKA$^{f/f}$ dams versus littermate control TetO-cre/AURKA$^{f/f}$ dams and representative H&E sections of mammary glands at 14 dL (2 dams per arm with 6 pups each; $n = 12$). DOX food was given from 1 dL to 14 dL (**d**) or from birth to 14 dL (**e**). Error bars represent mean ± s.e.m. Scale bars: 1 mm (whole-mount), and 100 μm (section).

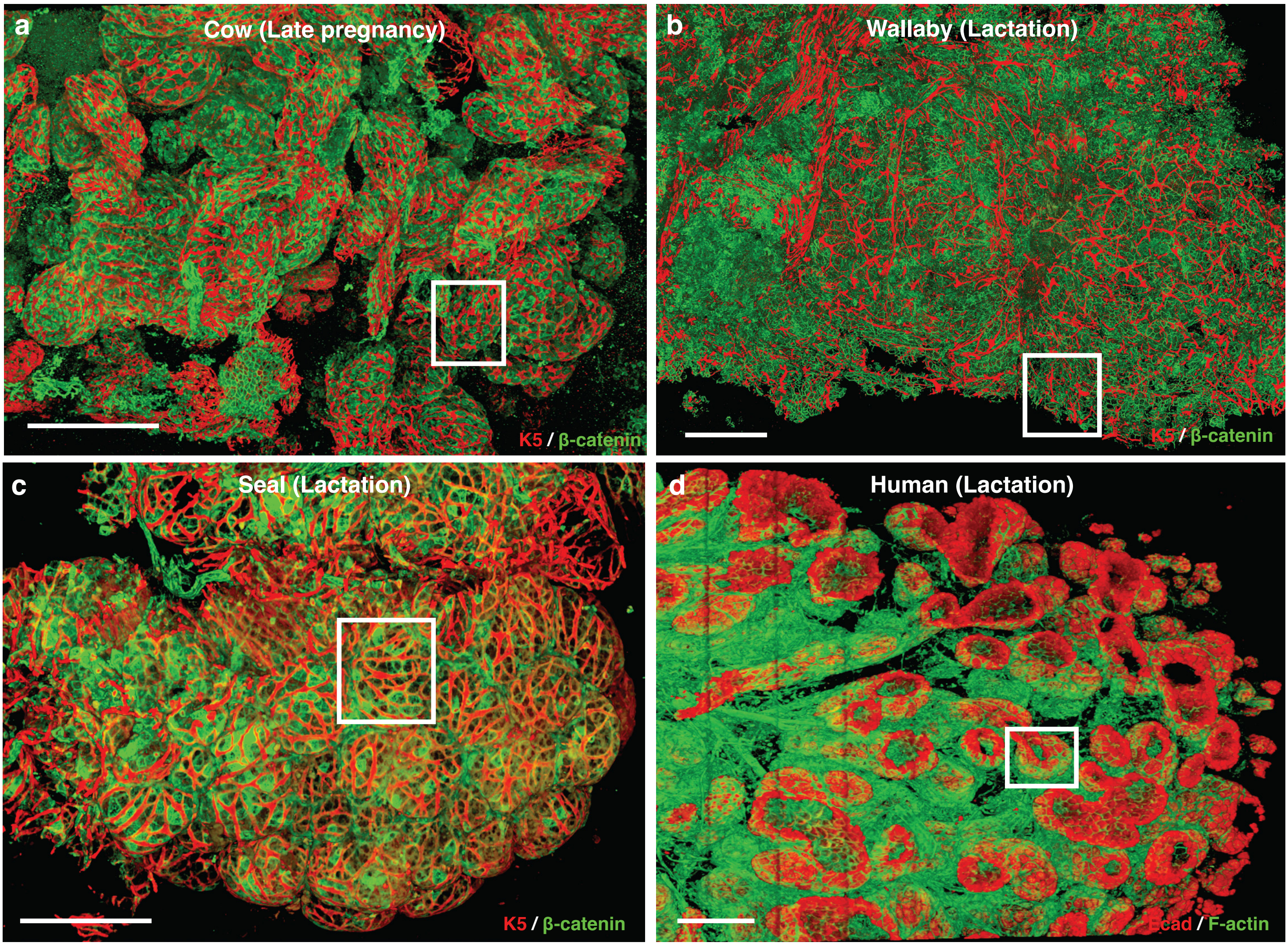

Supplementary Figure 10. Whole-mount images of lactating mammary glands from different species: cow, wallaby, seal and human

(**a**) Whole-mount 3D confocal image of a ductal portion from paraffin-embedded (PE) mammary tissue from a cow (n = 2, approximately 30 days prior to parturition). (**b**) Whole-mount 3D confocal image of a ductal portion from PE lactating mammary tissue from a wallaby (n = 3, all approximately 275 days lactation). (**c**) Whole-mount 3D confocal image of a ductal portion from PE lactating mammary tissue from a seal (n = 3, in mid-lactation at 5-6 months). (**d**) Whole-mount 3D confocal image of a ductal portion from fresh human lactating breast tissue (n = 5). The cow, seal and wallaby whole-mounts were labelled for Keratin 5 (red), β-catenin (green) and DAPI (white; not shown), whereas human whole-mounts were stained for E-cadherin (red), F-actin (green) and DAPI (white; not shown). The indicated enlargements are shown in Fig. 6. Scale bars: 200 μm.

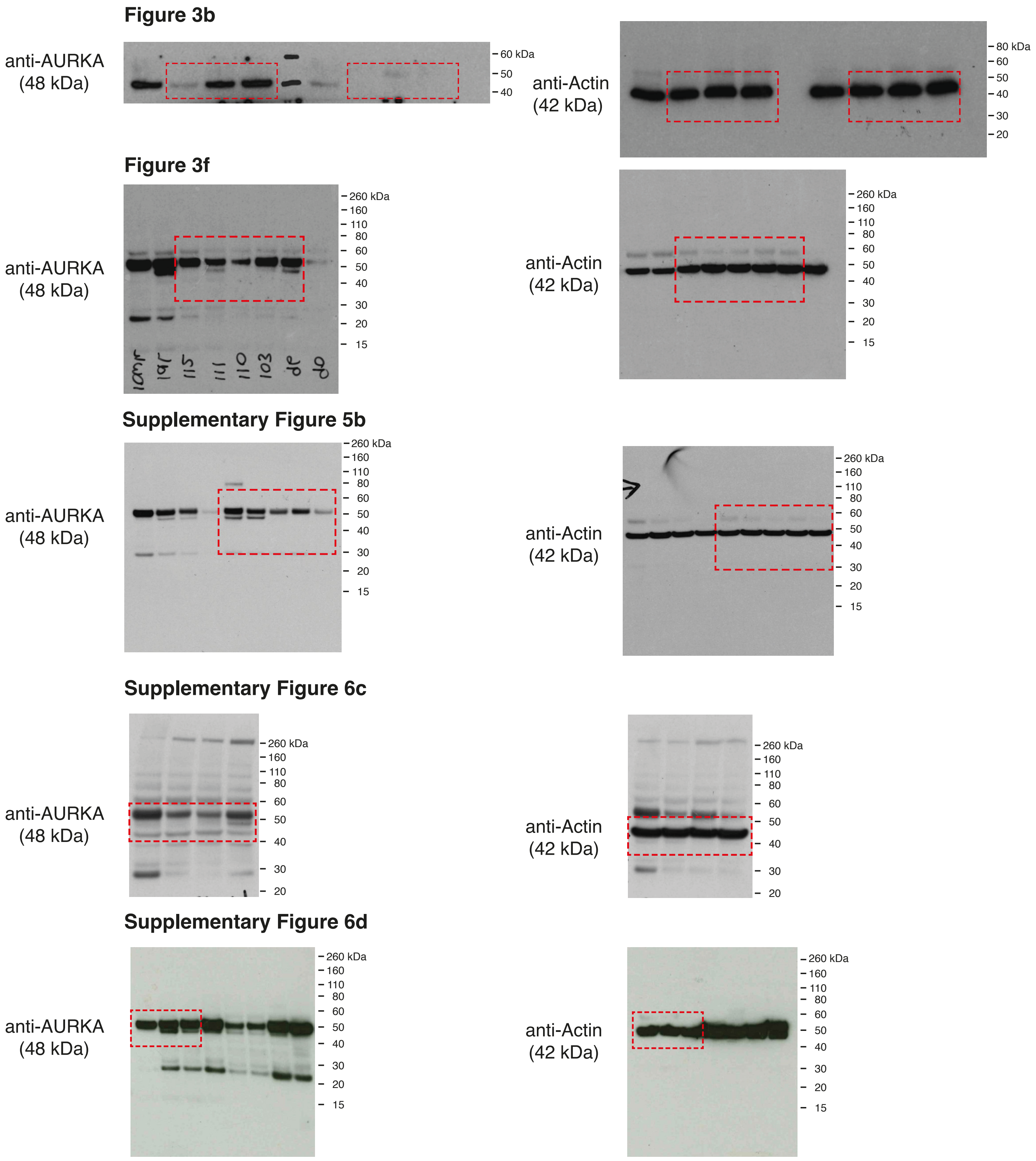

Supplementary Figure 11. Uncropped Western blots are shown for Figures 3b and 3f; Supplementary Figures 5b and 6c-d (representative of *n* = 2 or 3).
